# LncRNA CASC15, MiR-23b Cluster and SMAD3 form a Novel Positive Feedback Loop to promote Epithelial-Mesenchymal Transition and Metastasis in Ovarian Cancer

**DOI:** 10.7150/ijbs.67486

**Published:** 2022-02-21

**Authors:** Hui Lin, Xian Xu, Kelie Chen, Zhiqin Fu, Shengchao Wang, Yaqing Chen, Honghe Zhang, Yuequn Niu, Hanwen Chen, Hongfei Yu, Jian-zhong Shao, Weiguo Lu, Yihua Wu, Dajing Xia

**Affiliations:** 1 Department of Toxicology of School of Public Health, and Department of Gynecologic Oncology of Women's Hospital, Zhejiang University School of Medicine, Hangzhou, Zhejiang, 310058, China.; 2 Department of Gynecologic Oncology, Zhejiang Cancer Hospital, Hangzhou, Zhejiang, 310022, China.; 3 Department of Pathology, Key Laboratory of Disease Proteomics of Zhejiang Province, School of Medicine, Zhejiang University, Hangzhou, Zhejiang, 310058, China.; 4 Department of Gastroenterology, Second Affiliated Hospital, School of Medicine, Zhejiang University, Hangzhou, Zhejiang, 310009, China.; 5 College of Life Sciences, Key Laboratory for Cell and Gene Engineering of Zhejiang Province, Zhejiang University, Hangzhou, Zhejiang, 310058, China.

## Abstract

Cancer Susceptibility Candidate 15 (CASC15), which is a newly identified long noncoding RNA crucial for epigenetic regulation in human tumors, was found to be associated with poor prognosis of the patients with ovarian cancer by utilizing The Cancer Genome Atlas and Gene Expression Omnibus database. Therefore, the purpose of this paper was to explore the functional role and latent molecular mechanism of CASC15 in the progression of ovarian cancer. *In vitro* and *in vivo* experiments validated CASC15 as an oncogenic lncRNA in ovarian cancer, which could enhance metastasis through TGF-β-induced epithelial-mesenchymal transition progress. MiR-23b-3p and miR-24-3p, which are members of the miR-23b cluster, were identified to directly target CASC15 through luciferase assays. Further mechanistic investigations indicated that CASC15-mediated miR-23b-3p/miR-24-3p sequestration cooperatively upregulated SMAD3 expression, which, in turn, would permit increased CASC15 mRNA level as a transcription activation factor. This study first described a miR-23b-3p/miR-24-3p-mediated positive feedback loop between CASC15 and SMAD3, which may reflect the underlying molecular mechanism of CASC15's oncogenic function in ovarian cancer.

## Introduction

Ovarian cancer is one of the most malignant tumors in female reproductive organs, and it is the major cause of death related to gynecological cancer and the fifth-leading cause of overall cancer death among American women 1. Despite significant progress has been made in preventing, diagnosing and treating the ovarian cancer, lots of ovarian cancers remain to be diagnosed as advanced stages IV (29 percent) or III (51 percent) 2, 3, due to its usually symptomless early stages (I/II), meaning that the detected disease has usually metastasized within the abdomen. When the lesion is completely confined to one or both ovaries, the survival rate of five-year is 93 percent, but when the lesion spread in the abdominal cavity, the survival rate decreased to less than 30 percent. Therefore, the ratio of mortality to incidence is between 40 and 45 percent 1. Metastasis is a common characteristic of the advanced solid tumors, which represents the major cause of approximately 90 percent of cancer deaths 4. The transforming growth factor-β (TGF-β) signaling possesses a predominant effect on cancer metastasis, suppressing the growth of normal epithelial cells while promoting tumor cell metastasis via the tightly controlled and impactful epithelial-mesenchymal transition (EMT) process 5.

Noncoding RNAs, encompassing microRNA (miRNA) together with long noncoding RNA (lncRNA), are crucial for epigenetic regulation. Current evidence indicates that lncRNAs can employ as competitive endogenous RNAs (ceRNAs) for the competition of the microRNA binding sites to protect their target genes from degradation 6, 7, and some emerge as new players in tumor metastasis via modulating the EMT process. For instance, lncRNA linc00673 promoted TGF-β-induced EMT and cell metastasis by sponging miR-150-5p in non-small cell lung cancer 8. Another lncRNA PTAF has been found to promote TGF-β-induced EMT through the regulation of miR-25-SNAI2 axis in ovarian cancer 9. However, most of these studies focus on the interaction between one lncRNA and an individual miRNA 10-12, thus neglecting the fact that lncRNAs generally have multiple miRNA binding sites and can impair the activity of more than one miRNA or even a miRNA cluster through sequestration. In addition, the interaction between miRNA cluster and lncRNA is still largely unknown. In this paper, the potential for lncRNA Cancer Susceptibility Candidate 15 (CASC15) to sponge the miR-23b cluster was detected.

Recently, a novel oncogenic lncRNA, CASC15, is identified in various cancers such as basal cell 13, hepatic 14, 15 and tongue squamous cell carcinoma 16, as well as cervical 17, breast 18, melanoma 19-21, gastric 22, 23, colon 24, lung 25 and ovarian 26 cancer. Nevertheless, previously published studies on the effect of lncRNA CASC15 in ovarian cancer 26, 27 are not consistent. Moreover, although recent studies found that CASC15 not only enhanced cancer cell growth but also stimulated the progress of cancer metastasis via EMT 16, 20, 23, 24, the exact molecular mechanism underlying the oncogenic effect of CASC15 is still unclear. Therefore, further research is urgently required to explore the functional role and latent molecular mechanism of CASC15 in the progression of ovarian cancer. Our study identified an oncogenic role for lncRNA CASC15 in ovarian cancer. CASC15 promoted metastasis of ovarian cancer via the EMT progression partly through interaction with the miR-23b cluster. Further investigation suggested that CASC15 established a positive feedback loop for TGF-β/SMAD3 signaling: SMAD3 transcriptionally activated CASC15 expression, and CASC15 removed the miR-23b-3p/miR-24-3p suppression, thus upregulating the SMAD3 level. Our findings identified an epigenetic mechanism for CASC15-mediated cancer metastasis in ovarian cancer with implications in the diagnostic and therapeutic process.

## Materials and methods

### Chemicals and cell lines

Recombination human TGF-β1 (Cat. No.100-21, PeproTech, USA) was dissolved in10 mM citric acid (pH 3.0) supplement with 0.1% BSA to prepare a 10 ng/μl stock solution, and stored in aliquots at -20 °C. TGF-β1 receptor antagonist SB431542 (Cat. No. S1067, Selleckchem, China) was maintained as a stock solution (10 mM) at -20 °C.

Human ovarian adenocarcinoma (SKOV3 and OVCAR-3), human ovarian clear cell carcinoma (ES-2) cell lines and HEK-293T were provided by Cell Bank of the Chinese Academy of Sciences (Shanghai, China), the cell line of human ovarian adenocarcinoma A2780 was a gift from Dr. Hengyu Fan. The cell culture conditions employed in this paper see “Cell lines” in Supplementary Material.

### siRNA and miRNA transfection

Short interfering RNA (siRNA) against CASC15, SMAD3 or hepatocyte nuclear factor-1-β (HNF1B) and negative control as well as miRNA mimics, inhibitors and negative control were designed and subsequently produced by Genepharma (Shanghai, China). The siRNA oligonucleotide sequences were listed in Table S1.

### Constructs, transfection and viral infection

The full-length human CASC15 complementary DNA overexpression construct pcDNA3.1(-)-CASC15 was obtained from Loche (Hangzhou, China). The SMAD3 coding sequence was amplified using human complementary DNA as a template via PCR, subsequently, the PCR fragments were cloned into pcDNA3.1(-) plasmid (Invitrogen, USA).

An oligonucleotide sequence targeting CASC15 gene (sh-CASC15/sh-C2), which had the same target as si-C2, was cloned into pLKO.1-based lentiviral vector (Addgene, USA), and we used scramble shRNA (sh-NC) as the negative control. The sequences of hairpin were illustrated in Table S2.

SKOV3 cells stably transfected with shRNA were constructed in our laboratory. Lentivirus was packaged in HEK293 cells using standard protocols, and then SKOV3 cells were infected with viruses in medium containing polybrene (10 μg/ml, Sigma, USA), and selected with puromycin (1 μg/ml, Sangon Biotech, China).

For luciferase reporter assay, 3' untranslated region (UTR) fragment of CASC15, SMAD3 and HNF1B containing miR-23b cluster putative target sites were amplified and subsequently cloned downstream of the Firefly luciferase cassette in pmirGLO plasmid (Promega, USA). To measure promoter activities, sequential deletion fragments or full-length of human CASC15 and SMAD3 promoter were amplified using human genome DNA as a template via PCR, and cloned into pGL4.20 vector (Promega, USA). Besides, the constructs of CASC15-delete (-1397/-1385 delete) and SMAD3-delete (-1430/-1419 or -1387/-1375 delete) were obtained by inverse PCR using full-length pGL4.20-promoter as template, which deleted the putative binding sites. All sequences were confirmed by sequencing.

### Cell viability

The Cell Counting Kit-8 (CCK-8) assay (Beyotime, China) was employed to detect the cell viability in accordance with the protocol of manufacturer.

### Cell migration

Wound-healing assay was applied to evaluate cell migration. The details of wound-healing assay procedure see “Cell migration” in Supplementary Material.

### Cell invasion

Transwell assay was applied to evaluate cell invasion. The more information about transwell assay see “Cell invasion” in Supplementary Material.

### Western blots

The cells were harvested and lysed by addition of cell lysis buffer (Beyotime, China). The expression of EMT markers was assessed by western blots. The details of procedure and antibodies used see “Western blots” in Supplementary Material.

### Quantitative real-time PCR

We performed qPCR for detecting the expression levels of mRNA or microRNA. For details of qPCR procedures and specific primers, please refer to “Quantitative real-time PCR” in Supplementary Material and Table S3, S4, respectively.

### Luciferase reporter assay

3' UTR luciferase reporter assay was performed to predict and identify the interaction between CASC15/SMAD3/HNF1B and the miR-23b cluster. Promoter luciferase reporter assay was conducted for the measurement of the promoter activities at the transcriptional level. The details of luciferase reporter assay procedure see “Luciferase reporter assay” in Supplementary Material.

### *In vivo* tumor growth in mouse xenograft model

For *in vivo* experiments, the xenograft model using NOD/SCID mice was chosen as a well-established model of ovarian cancer (see “*In vivo* tumor growth in xenograft model” in Supplementary Material for more details).

### Statistical analyses

The statistical analyses were in accordance with our published research 28 (see “Statistical analyses” in Supplementary Material for more details).

## Results

### CASC15 was associated with poor survival in ovarian cancer

**Figure 1:**
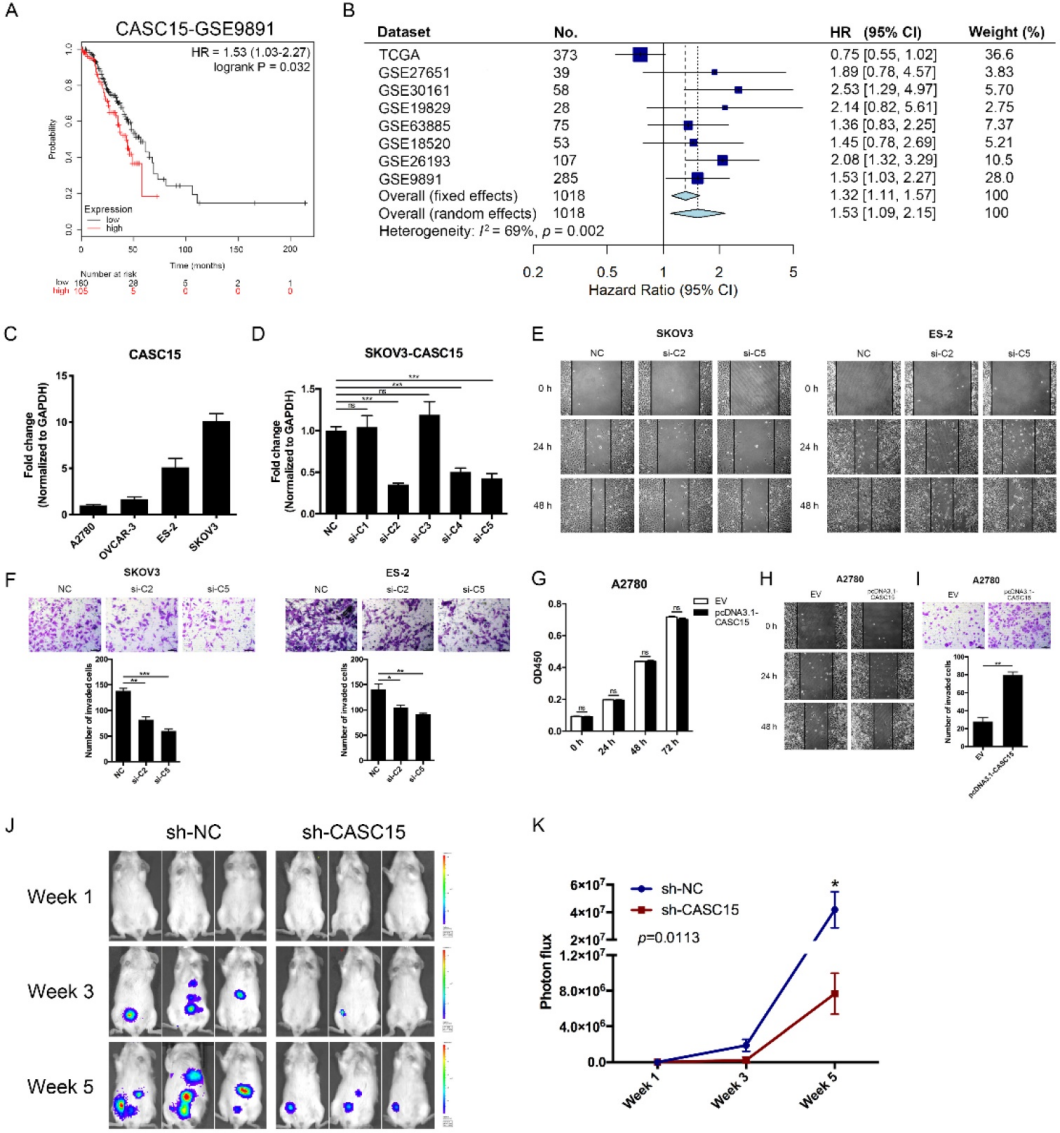
** CASC15 facilitated ovarian cancer cell migration and invasion *in vitro* and *in vivo*. (A)** Kaplan-Meier overall survival curves for ovarian cancer patients with high and low CASC15 expression from GEO database GSE9891. **(B)** Forest plot of hazard ratio (HR) for overall survival (OS) of ovarian cancer patients. **(C)** RT-qPCR analysis of CASC15 expression levels in 3 human ovarian adenocarcinoma cell lines (SKOV3, A2780 and OVCAR-3) and 1 human ovarian clear cell carcinoma cell line (ES-2). **(D)** Relative expression of CASC15 in SKOV3 cells 48 h post CASC15 siRNA transfection by RT-qPCR. **(E)** Representative images of the wound scratch assay captured under a light microscope (100×) using the SKOV3 or ES-2 cell line 24 and 48 h after scratching. Cells were transfected with si-NC or si-C2/si-C5. **(F)** Representative digital images (upper) and the number of invaded cells (lower) per 200× field of transwell invasion assay in SKOV3 and ES-2 cells after CASC15 knockdown. **(G)** CCK-8 proliferation assay in pcDNA3.1-CASC15 transfected A2780 cells. **(H)** Wound scratch assay in pcDNA3.1-CASC15 transfected A2780 cells. **(I)** Representative images (upper) and the number of invaded cells (lower) of transwell assay in A2780 cells. **(J)** Representative images of metastatic foci luciferase signals in NOD/SCID mice after i.p. injection of SKOV3 cells. **(K)** Quantitative analysis of total photon flux from metastatic foci. GAPDH serves as the internal control in RT-qPCR. Scale bar, 50 µM. ns, no statistical significance, * *p* <0.05, ** p <0.01, *** *p* <0.001. Values are mean ± SEM. Data are representative of three independent experiments.

The CASC15 expression data and corresponding clinical data of ovarian cancer were downloaded from the cancer genome atlas (TCGA) and gene expression omnibus (GEO) database. Using the Kaplan-Meier survival analysis in the GEO dataset GSE9891 29, GSE26193 30, we found that ovarian cancer patients with high CASC15 expression had worse overall survival (OS) than those with low CASC15 expression (Figure 1A and Figure S1A). 8 independent TCGA and GEO cohorts provided OS data (*n* = 1018 patients), and the hazard ratio (HR) and 95% confidence interval (CI) for each cohort and the summary HR are shown in Figure 1B 29-35. The overall summary estimated HR was 1.53 (95% CI: 1.09, 2.15, *p* = 0.014). Collectively, the data indicated that higher expression of CASC15 was associated with poor survival in ovarian cancer.

### CASC15 promoted ovarian cancer metastasis *in vitro* and *in vivo*

Four ovarian cancer cell lines were used in our study, including SKOV3, ES-2 with high expression of CASC15; and A2780, OVCAR-3 with low expression of CASC15 (Figure 1C). We transfected short interfering RNAs (siRNAs) specific for lncRNA CASC15 into SKOV3 and ES-2 cells (Figure 1D and Figure S1B). Lentiviral-based CASC15 stably depleted SKOV3 cells (pLKO.1-sh-CASC15 cells) were constructed via infecting a CASC15 specific shRNA (sh-CASC15/sh-C2) in our laboratory, which had the same target as si-C2 (Figure S1C). Moreover, to prevent the impact of siRNA off-target effect on observations, we not only used 2 independent siRNAs in this study but also transfected CASC15 overexpression vector (pcDNA3.1-CASC15) into A2780, ES-2 and OVCAR-3 cells, and CASC15 expression level was significantly elevated after pcDNA3.1-CASC15 transfection (Figure S1D).

The CCK-8 assay suggested that compared with the control group, CASC15 knockdown had no statistically significant effect on SKOV3 and ES-2 cell proliferation (Figure S1E, F). In parallel, CASC15 overexpression had no effect on A2780, ES-2 and OVCAR-3 cells either (Figure 1G and Figure S1G). What's more, we observed that inhibition of CASC15 expression strongly facilitated cisplatin-induced apoptosis in SKOV3 cells, as indicated by increased PARP and caspase-3 cleavage (Figure S1H).

We further evaluated whether CASC15 affected the migration and invasion of ovarian cancer cell *in vitro*. Wound scratch assay data demonstrated that CASC15 knockdown slowed wound closure in SKOV3 and ES-2 cells (Figure 1E and Figure S1I). Furthermore, after CASC15 specific siRNA transfection or shRNA stable infection, transwell invasion assay showed significantly fewer invaded cells in SKOV3 and ES-2 cells compared with the corresponding control group (Figure 1F and Figure S1I). To further support these results, we transfected pcDNA3.1-CASC15 into A2780 (Figure 1H, I), ES-2 and OVCAR-3 (Figure S1J, K) cells, which also showed increased migration and invasion ability in the wound scratch and transwell assays.

To further verify the pro-metastatic role of CASC15 in ovarian cancer *in vivo*, NOD/SCID mice were intraperitoneally injected with SKOV3 cells stably transfected with sh-CASC15 or sh-NC shRNA, and then to monitor metastasis, the mice were subjected to bioluminescent imaging on week 1, 3, and 5. After 5 weeks in the CASC15 knockdown group, the whole-body luminescence signal was approximately 6 times lower than that in the control group (Figure 1J, K). Moreover, CASC15 knockdown remarkably decreased the metastatic nodule number within the abdominal cavity (Figure 1J). In conclusion, results obtained in this mouse model strongly supported the role of CASC15 as a metastatic promoter in ovarian cancer.

### CASC15 promoted EMT via TGF-β/SMAD3 pathway in ovarian cancer cells

**Figure 2:**
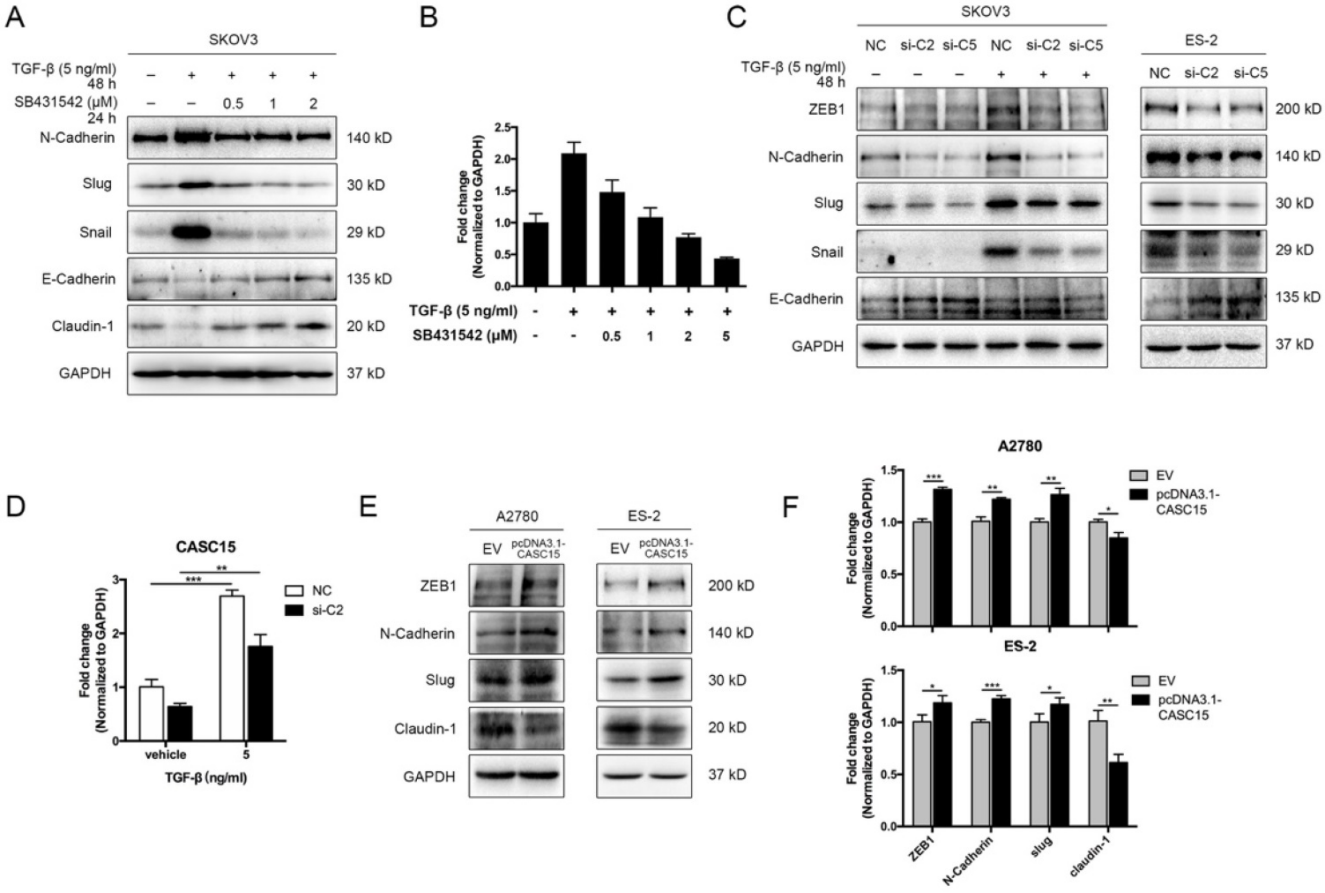
** CASC15 was upregulated by TGF-β and triggered EMT in ovarian cancer cells. (A)** Western blots of N-Cadherin, Slug, Snail, E-Cadherin, and Claudin-1 in TGF-β and SB431542 treated SKOV3 cells. **(B)** Expression of CASC15 in TGF-β and SB431542 treated SKOV3 cells by RT-qPCR. **(C)** Western blots of EMT markers in SKOV3 and ES-2 cells with si-NC or si-C2/si-C5 transfection. **(D)** CASC15 mRNA level in si-NC or si-C2 transfected, TGF-β treated SKOV3 cells by RT-qPCR. **(E)** Western blots of ZEB1, N-Cadherin, Slug and Claudin-1 in pcDNA3.1-CASC15 transfected A2780 and ES-2 cells. **(F)** RT-qPCR analysis of EMT markers in pcDNA3.1-CASC15 transfected A2780 and ES-2 cells. GAPDH serves as the internal control in RT-qPCR and western blots. ns, no statistical significance, * *p* <0.05, ** p <0.01, *** *p* <0.001. Values are mean ± SEM. Data are representative of three independent experiments.

We adopted TGF-β1 as an EMT inducer in the following experiments. In order to detect the EMT process, the expression levels of EMT markers were assessed by western blots, including epithelial markers (E-cadherin and Claudin-1) and mesenchymal markers (ZEB1, N-Cadherin, Slug, and Snail), morphological examination was conducted as well. In SKOV3 cells, TGF-β1 (5 ng/mL) was confirmed to be sufficient to trigger EMT progression (Figure S2A, B), while the antagonist of TGF-β receptor SB431542 could block the phenotypes of EMT in a dose-dependent manner (Figure 2A). Interestingly, RT-qPCR data indicated that CASC15 expression was remarkably elevated under the TGF-β1 stimulation and could be rescued by SB431542 (Figure 2B). However, according to western blots of EMT markers and morphological features, ES-2, A2780 and OVCAR-3 cells were relatively insensitive to TGF-β1 (Figure S2B, C).

Since CASC15 was upregulated via TGF-β1, we tried to detect whether CASC15 regulated the EMT program in ovarian cancer. The correlation between CASC15 and EMT-related genes was subsequently studied via RT-qPCR and western blotting. We observed that CASC15 knockdown could enhance E-Cadherin expression and reduce ZEB1, N-Cadherin, Slug, and Snail expression (Figure 2C, D and Figure S2D-F) in SKOV3 and ES-2 cells. Consistently, as illustrated in Figure 2E, F and Figure S2G, the excessive expression of CASC15 led to a significant decrease in Claudin-1 and increases in ZEB1, N-Cadherin and Slug expression levels.

**Figure 3:**
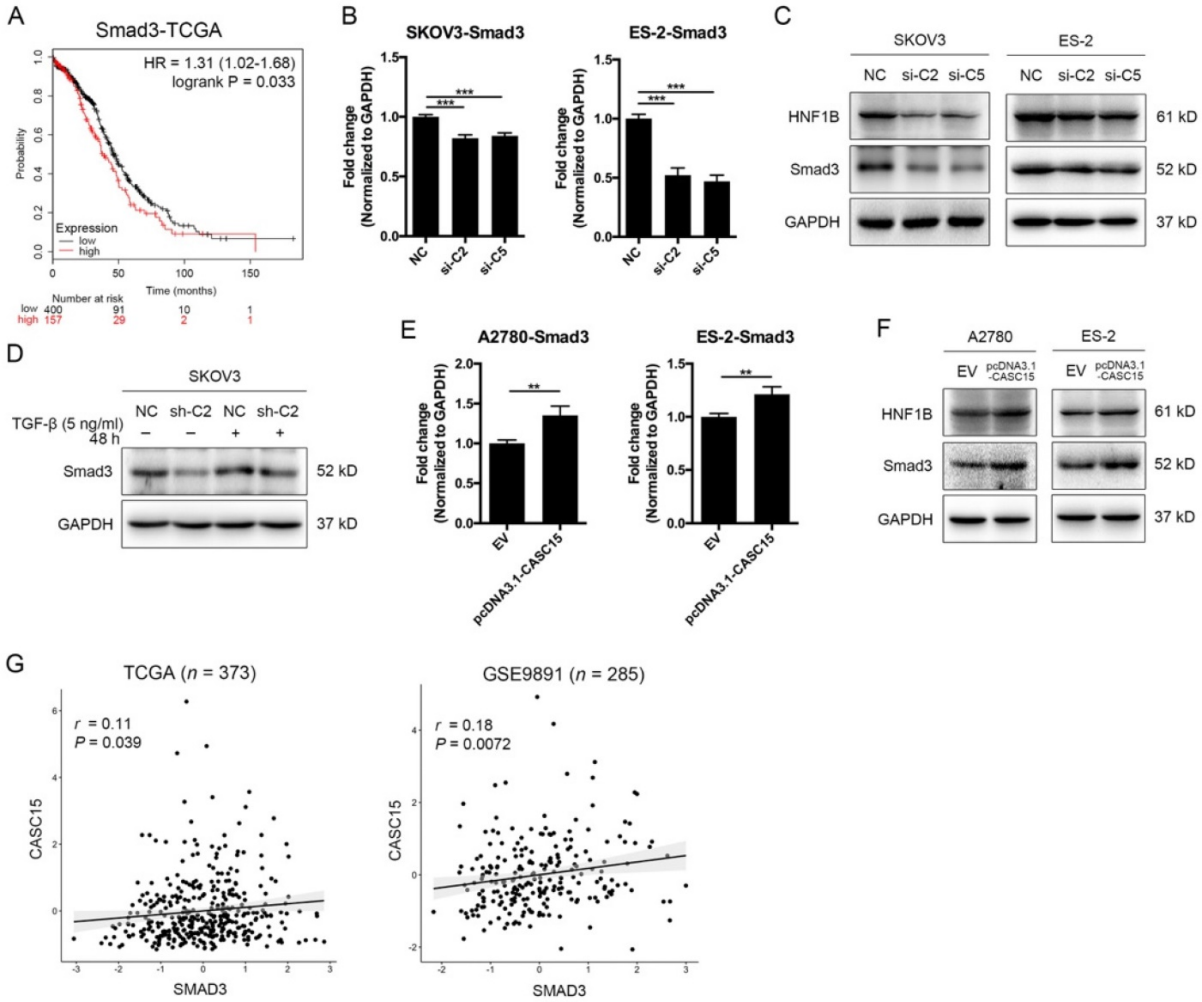
** Positive correlations between CASC15 and SMAD3/HNF1B. (A)** Kaplan-Meier overall survival curves for ovarian cancer patients with high and low SMAD3 expression from the TCGA database. **(B)** RT-qPCR analysis of SMAD3 expression in si-NC or si-C2/si-C5 transfected SKOV3 and ES-2 cells. **(C)** Western blots of SMAD3 and HNF1B in si-NC or si-C2/si-C5 transfected SKOV3 and ES-2 cells. **(D)** Western blots of SMAD3 expression in pLKO.1-sh-CASC15 and pLKO.1-sh-NC transfected SKOV3 cells after TGF-β or vehicle treatment. **(E)** RT-qPCR analysis of SMAD3 expression in pcDNA3.1-CASC15 transfected A2780 and ES-2 cells. **(F)** Western blots of SMAD3 and HNF1B in pcDNA3.1-CASC15 transfected A2780 and ES-2 cells. **(G)** Scatter plots of CASC15 vs. SMAD3 expression in the TCGA and GEO database. *p* values and pearson correlation coefficients (*r*) are shown. GAPDH serves as the internal control in RT-qPCR and western blots. ns, no statistical significance, * *p* <0.05, ** p <0.01, *** *p* <0.001. Values are mean ± SEM. Data are representative of three independent experiments.

As described above, CASC15 possessed a significant effect on the EMT program and metastasis in ovarian cancer, but the specific mechanism of CASC15 promoting EMT process remains unclear. SMAD3 is a well-acknowledged critical transcription factor in the signaling pathway of TGF-β 36 and also a latent biomarker for ovarian cancer prognosis prediction (Figure 3A). Remarkably, as shown in Figure 3B-D, knockdown of CASC15 led to an evident reduction in the protein and mRNA levels of SMAD3 in SKOV3 and ES-2 cells; following CASC15 overexpression in A2780 and ES-2 cells, the expression of SMAD3 increased (Figure 3E, F). Analysis of TCGA and GEO data repository exhibited that SMAD3 expression was positively correlated with the expression of CASC15 (Figure 3G), suggesting that CASC15 promoted the EMT program in a SMAD3-dependent manner.

### Reciprocal correlation between CASC15 and miR-23b-3p/ miR-24-3p

**Figure 4:**
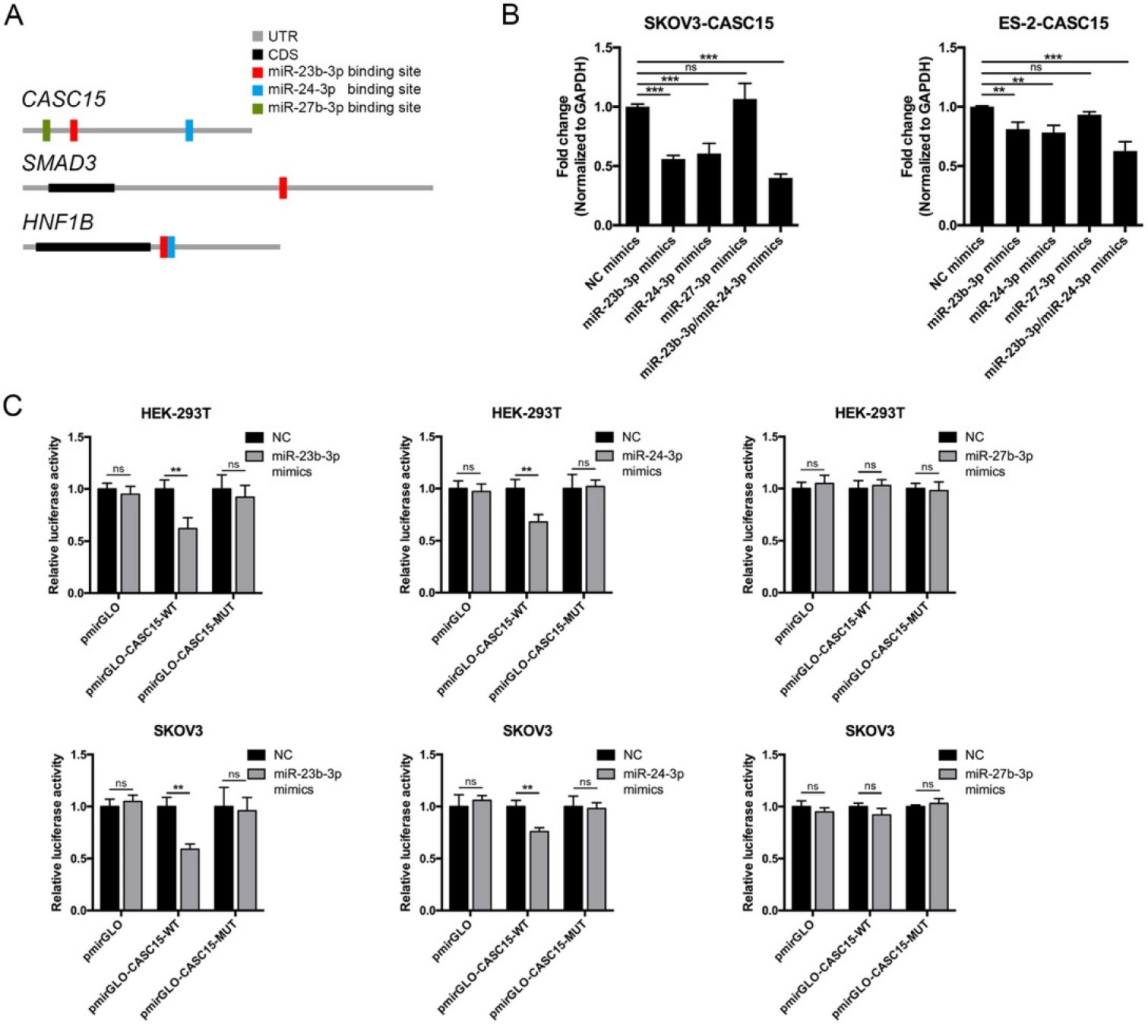
** Reciprocal correlation between CASC15 and miR-23b-3p/miR-24-3p. (A)** Predicted miR-23b-3p (red), miR-24-3p (blue), and miR-27b-3p (green) binding sites in 3' UTRs of human *CASC15*, *SMAD3* and *HNF1B* mRNAs. **(B)** RT-qPCR analysis of CASC15 mRNA expression in SKOV3 and ES-2 cells 48 h post transfection with miR-23b-3p, miR-24-3p, and miR-27b-3p mimics alone, or miR-23b-3p and miR-24-3p mimics together. **(C)** Relative luciferase activity in HEK-293T and SKOV3 cells cotransfected with miR-23b-3p, miR-24-3p, or miR-27b-3p mimics and control or reporter luciferase plasmids containing the full-length CASC15. GAPDH serves as the internal control in RT-qPCR. ns, no statistical significance, * *p* <0.05, ** p <0.01, *** *p* <0.001. Values are mean ± SEM. Data are representative of three independent experiments.

To explore the function of CASC15 in ovarian cancer, the online bioinformatics tool DIANA-LncBase v.2 was used to find the experimentally supported and *in silico* predicted candidate miRNA recognition elements (MREs) on CASC15 37, and the DIANA data indicated that miR-23b cluster, comprising miR-23b-3p, miR-24-3p and miR-27b-3p, might bind to CASC15 in a complementary manner (Figure 4A and Table S5). Moreover, Rogler et al. reported that the miRNAs of miR-23b cluster target SMAD3, resulting in a block of TGF-β family signaling 38, suggesting that the association might be important for CASC15-induced EMT.

Thus, miR-23b cluster miRNA mimics were transfected into SKOV3 and ES-2 cells, and RT-qPCR data showed that CASC15 expression could be negatively regulated by miR-23b-3p and miR-24-3p, but not miR-27b-3p and that the decrease was greater in the cells transfected by both miR-23b-3p and miR-24-3p mimics (Figure 4B). Using dual-luciferase reporter assays, we confirmed that lncRNA CASC15 was the direct target of miR-23b-3p and miR-24-3p, but not miR-27b-3p, in both HEK-293T and SKOV3 cells, and that repression of luciferase activity was rescued by mutations in corresponding sites (Figure 4C). Therefore, the following study focused on the interaction between CASC15 and miR-23b-3p/miR-24-3p.

### MiR-23b-3p and miR-24-3p repressed SMAD3 directly and indirectly through transcription factor HNF1B

**Figure 5:**
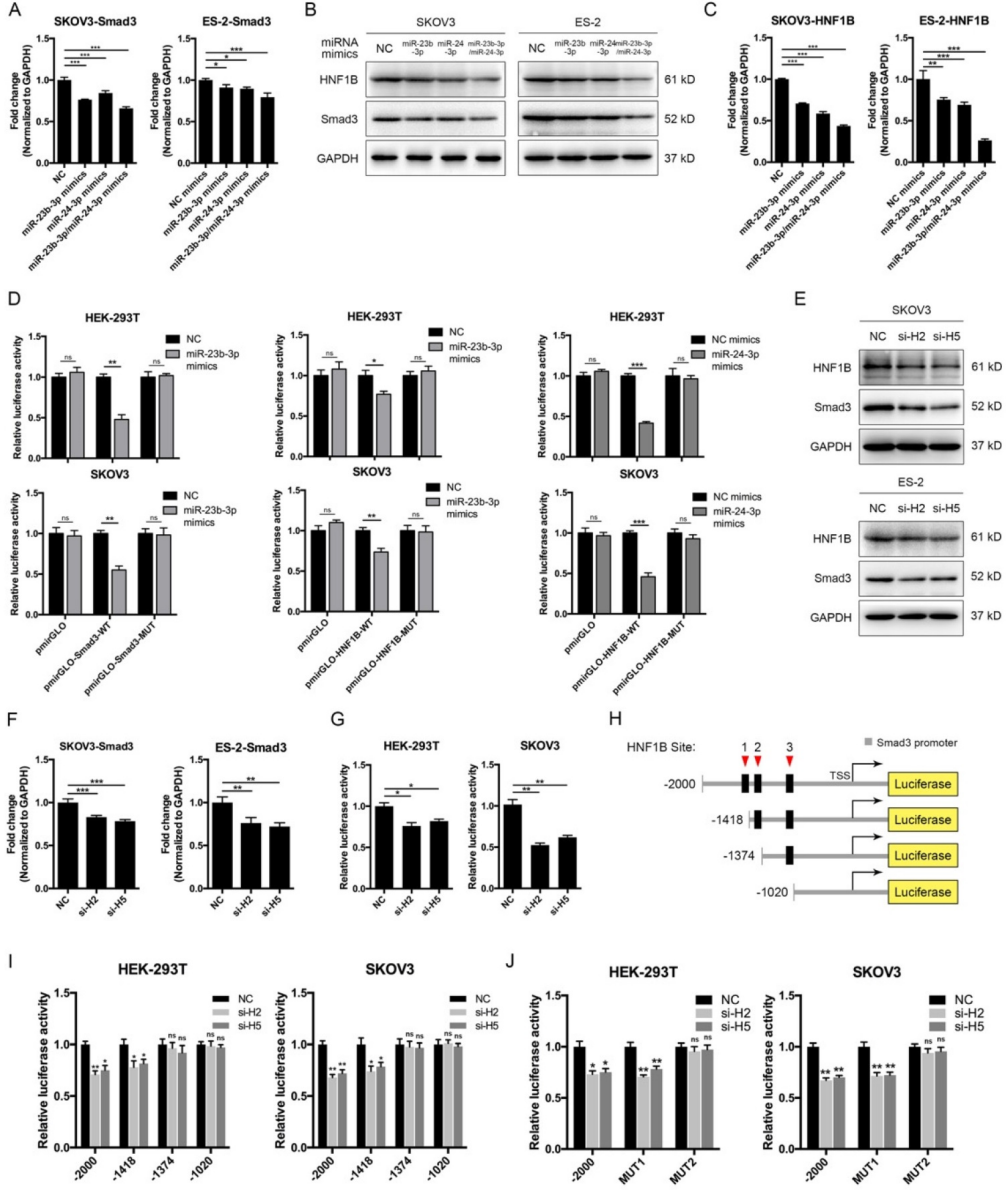
** MiR-23b-3p and miR-24-3p repressed SMAD3 directly, and indirectly through transcription factor HNF1B. (A)** RT-qPCR analysis of the expression of SMAD3 in SKOV3 and ES-2 cells 48 h post transfection with miR-23b-3p or miR-24-3p mimics alone, or miR-23b-3p and miR-24-3p mimics together. **(B)** Western blots of SMAD3 and HNF1B in SKOV3 and ES-2 cells 48 h post transfection with miR-23b-3p or miR-24-3p mimics alone, or miR-23b-3p and miR-24-3p mimics together. **(C)** RT-qPCR analysis of HNF1B expression in miR-23b-3p or miR-24-3p mimics-transfected SKOV3 and ES-2 cells. **(D)** Relative luciferase activity in HEK-293T and SKOV3 cells cotransfected with miR-23b-3p or miR-24-3p mimics and control or reporter luciferase plasmids containing the HNF1B or SMAD3 3' UTR. **(E)** Western blots of SMAD3 and HNF1B in si-NC or si-H2/si-H5 transfected SKOV3 and ES-2 cells. **(F)** RT-qPCR analysis of SMAD3 expression in si-NC or si-H2/si-H5 transfected SKOV3 and ES-2 cells. **(G)** Luciferase reporter assays were conducted by cotransfection of pGL4.20-SMAD3 promoter luciferase reporter with si-NC or si-H2/si-H5, as well as a *Renilla* luciferase reporter in HEK-293T and SKOV3 cells. **(H)** The schematic diagrams of deletion constructs between the -2,000 to +500 region of the SMAD3 promoter. **(I)** The luciferase vector pGL4.20 containing full-length or sequential deletion of SMAD3 promoter was transfected in HEK-293T and SKOV3 cells, and the luciferase activity was detected. **(J)** The luciferase vector pGL4.20 containing wild-type or mutant (MUT) SMAD3 promoter was transfected in HEK-293T and SKOV3 cells, and the luciferase activity was detected. GAPDH serves as the internal control in RT-qPCR and western blots. ns, no statistical significance, * *p* <0.05, ** p <0.01, *** *p* <0.001. Values are mean ± SEM. Data are representative of three independent experiments.

The above results showed that CASC15 promoted EMT via the TGF-β/SMAD3 pathway, and the miR-23b cluster was revealed to target SMAD3 in liver stem cells 38. However, using the computational target prediction tool DIANA-microT-CDS 39, we found that the 3' UTR of SMAD3 was targeted by miR-23b-3p, but not by miR-24-3p (Figure 4A and Table S5). Given that miR-23b-3p and miR-24-3p alone or in combination could all significantly repress SMAD3 expression in SKOV3 and ES-2 cells (Figure 5A, B), we supposed that miR-24-3p might reduce the expression of SMAD3 indirectly through a positive transcription factor which contains binding sites in the 2.5 kb promoter of SMAD3. Therefore, the online programs PROMO (TRANSFAC v8.3) and JASPAR were applied for the prediction of transcription factor binding sites in the SMAD3 promoter, and the transcription factor data were overlapped with miR-24-3p target prediction results of TargetScan, DIANA and starBase v2.0 tools (Figure 4A and Table S5). Ultimately, the transcription factor hepatocyte nuclear factor-1-β (HNF1B), which is closely related to ovarian cancer risk 40, 41, drew our attention. Furthermore, HNF1B was downregulated in SKOV3 and ES-2 cells after CASC15 knockdown (Figure 3C and Figure S3A), while it was upregulated in A2780 and ES-2 cells after CASC15 overexpression (Figure 3F and Figure S3B).

Of note, DIANA results showed that the 3' UTR of HNF1B included not only predicted miR-24-3p but also miR-23b-3p targeting sites (Figure 4A and Table S5), and western blot and RT-qPCR data indicated that miR-24-3p and miR-23b-3p repressed HNF1B expression cooperatively (Figure 5B, C). Moreover, the 3' UTR luciferase reporter assay in HEK-293T and SKOV3 cells verified the miR-23b-3p binding sites on the 3' UTR of SMAD3 and HNF1B mRNA, and miR-24-3p binding elements on the HNF1B mRNA, while mutations in these binding sites reversed luciferase activity (Figure 5D).

To confirm whether HNF1B would regulate the transcriptional activity of SMAD3, HNF1B specific siRNAs were transfected into SKOV3 and ES-2 cells (Figure 5E and Figure S3C), and HNF1B knockdown significantly decreased SMAD3 expression at the transcriptional and translational levels (Figure 5E, F), revealing a promoting effect of HNF1B on SMAD3. The luciferase reporter assay suggested that HNF1B knockdown resulted in an inhibition of SMAD3 promoter activity (Figure 5G). Three putative HNF1B binding elements were found in the SMAD3 promoter region by the bioinformatics tools PROMO and JASPAR (Figure 5H, Figure S4A and Table S6). The SMAD3 promoter was truncated to remove regions with which the HNF1B may interact in order to determine whether the HNF1B-binding regions are functional. Serial deletion as well as mutation constructs of the SMAD3 promoter were generated in our laboratory (Figure 5H). The luciferase activity was significantly repressed after HNF1B siRNA transfection when the construct contained the SMAD3 promoter region of -1418/+500, but not -1374/+500 or -1020/+500 (Figure 5I). Mutations of HNF1B-binding sites 1 and 2 indicated that HNF1B-binding site 2 (-1387/-1375 upstream of transcription start site, TSS) was the main position for HNF1B stimulation of SMAD3 transcription activity (Figure 5J).

### MiR-23b-3p and miR-24-3p additively suppressed metastasis, and rescued CASC15's promoting effect

**Figure 6:**
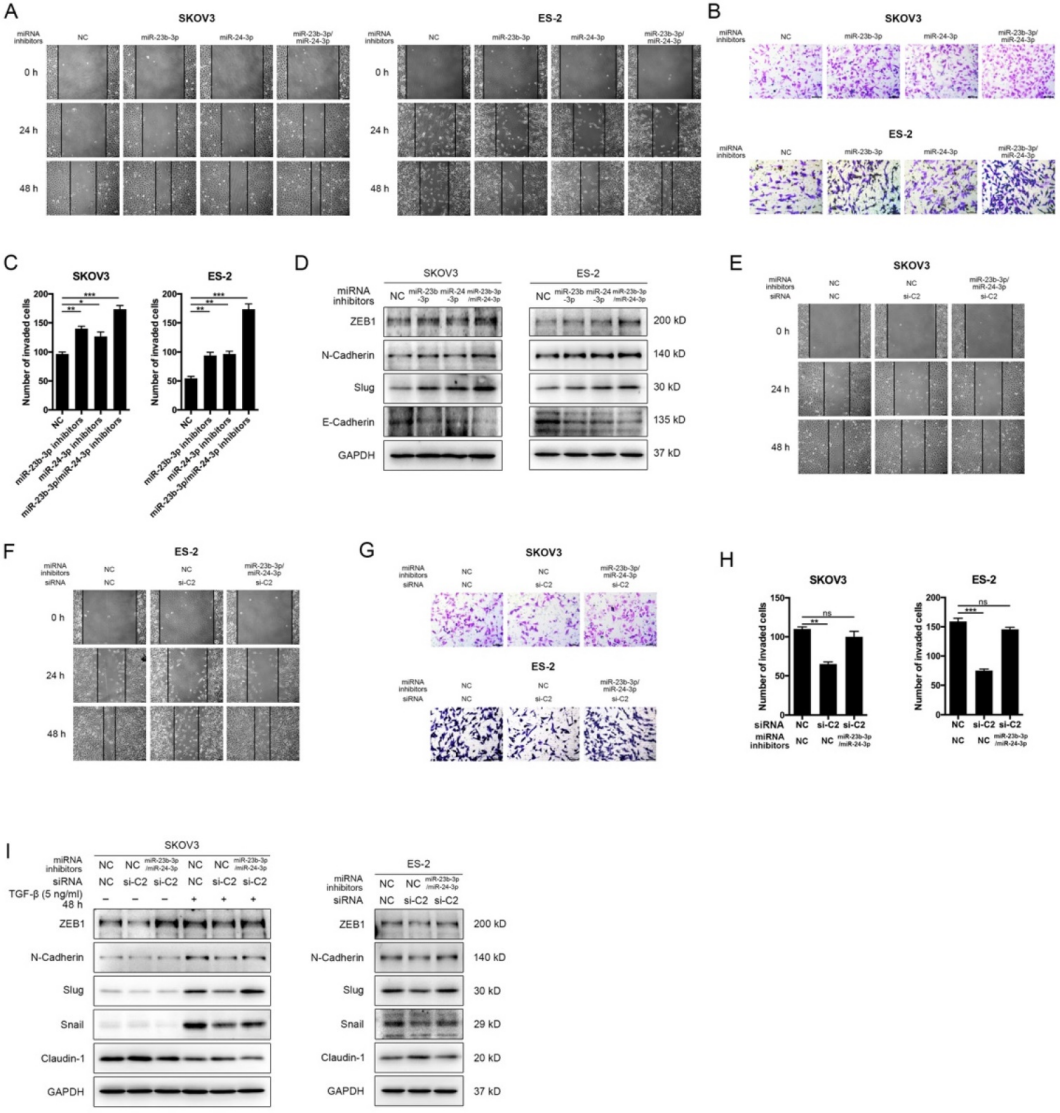
** MiR-23b-3p and miR-24-3p additively suppressed EMT and metastasis, and rescued CASC15's promoting effect on ovarian cancer cells. (A)** Representative images of the wound scratch assay captured under a light microscope (100×) using the SKOV3 or ES-2 cell line 24 and 48 h after scratching. Cells were transfected with miR-23b-3p or miR-24-3p inhibitors alone, or miR-23b-3p and miR-24-3p inhibitors together. **(B and C)** Representative digital images (B) and quantitative analysis (C) of transwell invasion assay in SKOV3 and ES-2 cells after transfection with miR-23b-3p or miR-24-3p inhibitors alone, or miR-23b-3p and miR-24-3p inhibitors together. **(D)** Western blots of EMT markers in SKOV3 and ES-2 cells after transfection with miR-23b-3p or miR-24-3p inhibitors alone, or miR-23b-3p and miR-24-3p inhibitors together. **(E)** Wound scratch assay in miR-23b-3p/miR-24-3p inhibited and si-NC/si-C2 transfected SKOV3 cells. **(F)** Wound scratch assay in miR-23b-3p/miR-24-3p inhibited and si-NC/si-C2 transfected ES-2 cells. **(G)** Transwell invasion assay in miR-23b-3p/miR-24-3p inhibited and si-NC/si-C2 transfected SKOV3 and ES-2 cells. **(H)** The quantitative analysis of invaded cell number in miR-23b-3p/miR-24-3p inhibited and si-NC/si-C2 transfected SKOV3 and ES-2 cells. **(I)** Western blots of EMT markers in miR-23b-3p/miR-24-3p inhibited and si-NC/si-C2 transfected SKOV3 and ES-2 cells. GAPDH serves as the internal control in RT-qPCR and western blots. Scale bar, 50 µM. ns, no statistical significance, * *p* <0.05, ** p <0.01, *** *p* <0.001. Values are mean ± SEM. Data are representative of three independent experiments.

To determine whether miR-23b-3p and miR-24-3p were participated in the EMT progress and cancer metastasis, miR-23b-3p and miR-24-3p inhibitors were transfected individually or together into SKOV3, ES-2 and OVCAR-3 cells. The wound scratch and transwell assays indicated that individual miR-23b-3p and miR-24-3p inhibitors significantly increased migration and invasion ability respectively in 3 cell lines, whereas the increment in metastasis ability was more pronounced with combined treatment (Figure 6A-C and Figure S3D, E). Moreover, inhibition of miR-23b-3p and miR-24-3p resulted in a remarkable reduction in E-Cadherin as well as increases in the ZEB1, N-Cadherin, and Slug expression in SKOV3, ES-2 and OVCAR-3 cells, and those miRNAs exhibited additive effects (Figure 6D and Figure S3F, G).

We then investigated the influence of CASC15 on cell migration, invasion and EMT after miR-23b-3p and miR-24-3p were restrained to validate the interaction between CASC15 and miR-23b-3p/miR-24-3p. The experimental data showed that, in consistent with previous results, the knockdown of CASC15 significantly reduced the migration and invasion abilities of SKOV3 and ES-2 cells and that reduction would be partly abolished when miR-23b-3p and miR-24-3p inhibitors were transfected in combination (Figure 6E-H), Furthermore, miR-23b-3p and miR-24-3p inhibition in SKOV3 and ES-2 cells opposed CASC15 knockdown-mediated Claudin-1 upregulation as well as ZEB1, N-Cadherin, Slug and Snail downregulation (Figure 6I).

### SMAD3 activated CASC15 transcription and formed a novel positive feedback loop

**Figure 7:**
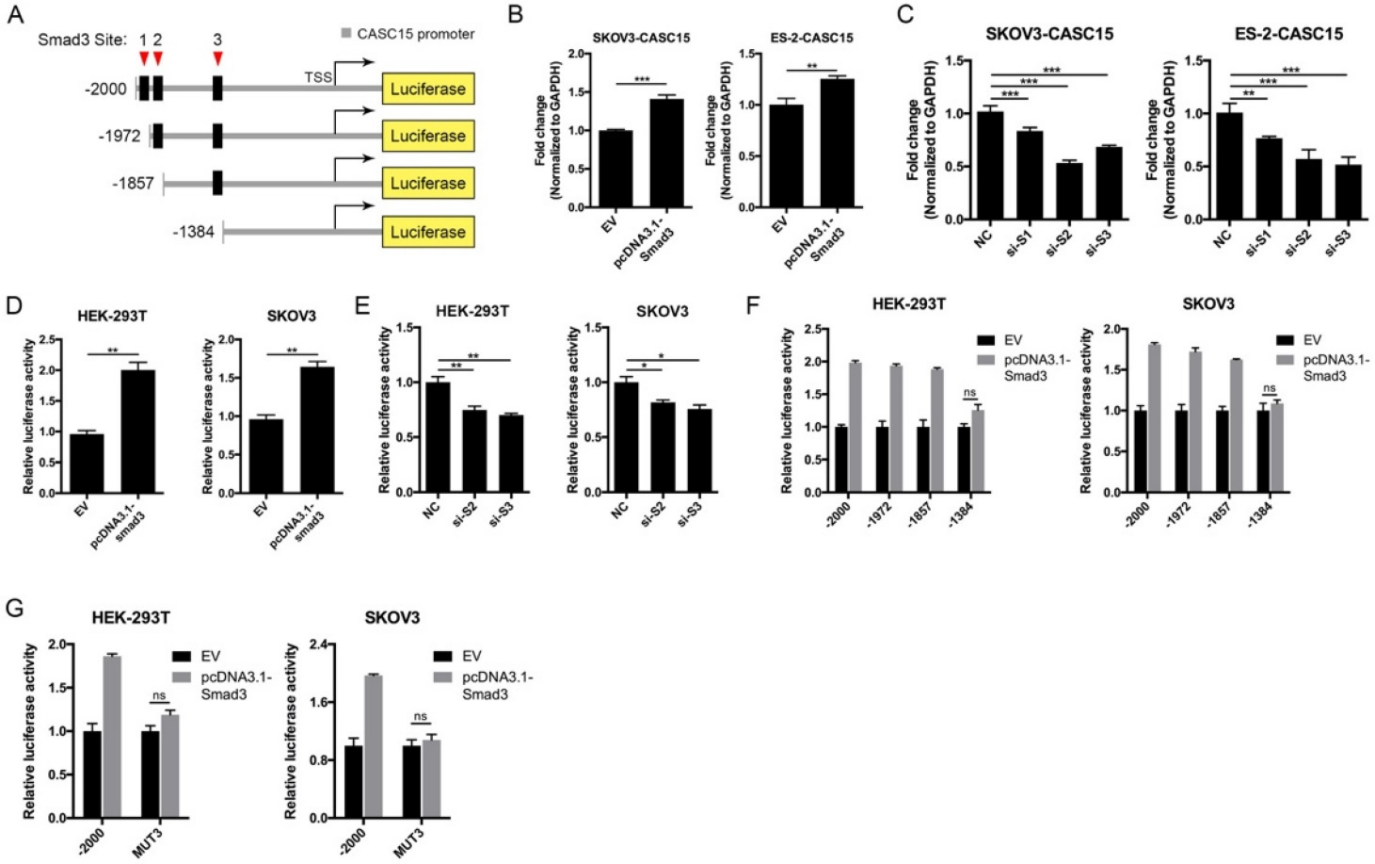
** SMAD3 activated CASC15 transcription and formed a novel positive feedback loop. (A)** The schematic diagrams of deletion constructs between the -2,000 to +500 region of the CASC15 promoter. The predicted SMAD3-binding sites in the promoter are represented in black boxes. **(B)** RT-qPCR analysis of the expression of CASC15 in pcDNA3.1-SMAD3 transfected SKOV3 and ES-2 cells. **(C)** RT-qPCR analysis of the expression of CASC15 in SMAD3 specific siRNAs transfected SKOV3 and ES-2 cells. **(D)** The relative luciferase activity of CASC15 in HEK-293T or SKOV3 cell line with or without SMAD3 overexpression when cotransfected with pGL4.20-CASC15 promoter luciferase reporter and pRL-TK *Renilla* luciferase control reporter. **(E)** The relative luciferase activity of pGL4.20-CASC15 promoter reporter in HEK-293T and SKOV3 cells with si-NC or si-S2/si-S3 transfection. **(F)** The luciferase vector pGL4.20 driven by full-length or sequential deletion of CASC15 promoter was transfected in HEK-293T and SKOV3 cells, and luciferase activity was measured. **(G)** The luciferase vector pGL4.20 containing wild-type or mutant (MUT) CASC15 promoter was transfected in HEK-293T and SKOV3 cells, and the luciferase activity was detected. GAPDH serves as the internal control in RT-qPCR. ns, no statistical significance, * *p* <0.05, ** p <0.01, *** *p* <0.001. Values are mean ± SEM. Data are representative of three independent experiments.

**Figure 8:**
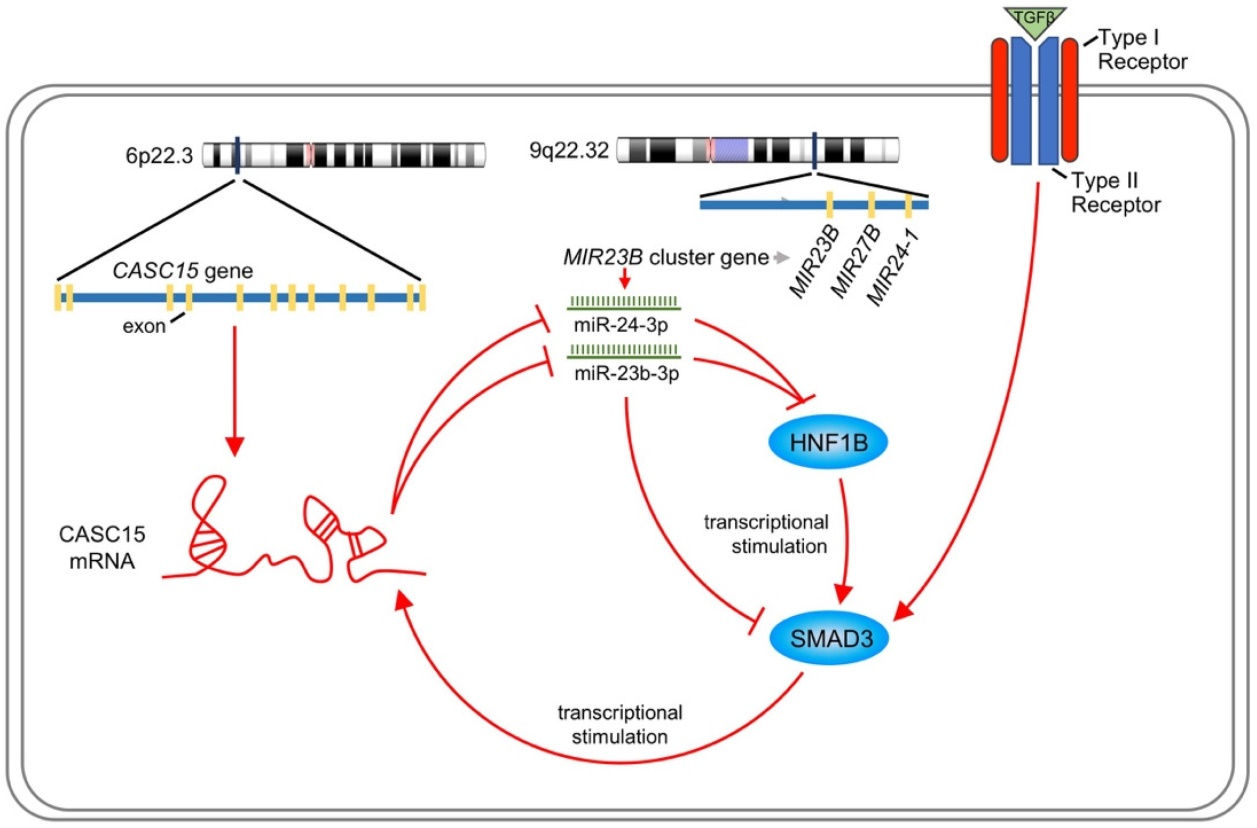
Schematic plot illustrating that CASC15 formed a new positive feedback loop with SMAD3.

Like most transcription factors, SMAD3 recognizes a specific dsDNA sequence known as the SMAD3 binding element (SBE). Interestingly, JASPAR results showed that the 2.5 kb promoter of CASC15 contained 3 SMAD3 binding sites (Figure 7A, Figure S4B and Table S7). CASC15 mRNA was significantly increased in SMAD3-overexpressed SKOV3 and ES-2 cells (Figure 7B), while SMAD3 knockdown resulted in CASC15 downregulation (Figure 7C and Figure S4C), indicating that SMAD3 could promote CASC15 transcription. Moreover, luciferase reporter assays indicated that overexpression of SMAD3 markedly upregulated CASC15 promoter activity in HEK-293T and SKOV3 cells, while knockdown of SMAD3 led to a corresponding reduction (Figure 7D, E). As illustrated in Figure 7A, the constructs of sequential deletions and mutations of SMAD3 binding sites were generated in our laboratory, and we examined the activities of those promoter fragments fused to the luciferase gene. After co-transfecting with SMAD3 overexpression vector into HEK-293T and SKOV3 cells, the luciferase activity of the -1384/+500 reporter exhibited no obvious change (Figure 7F), demonstrating that the position of -1857/-1385 contained the crucial SMAD3 binding element in the CASC15 promoter. In addition, the mutation of SMAD3-binding site 3 (-1397/-1385 upstream of TSS) significantly abolished luciferase activity induced by SMAD3 (Figure 7G). Taken together, the present findings of our study suggested that a double-negative, namely, positive, regulatory circuit among CASC15 and SMAD3 may underlie CASC15-mediated ovarian cancer cell EMT and metastasis induction (Figure 8).

## Discussion

The* CASC15* gene locus was initially predicted by bioinformatics method and experimentally verified between the *SOX4* and *PRL* genes on human chr6 42. Afterwards, Maris et al. 43 performed an independent genome-wide association study, identifying that the *CASC15* locus is at chr6p22, within which three single nucleotide polymorphisms (SNPs) were related to the susceptibility to neuroblastoma. More recently, several researches have suggested that the lncRNA CASC15 has oncogenic effects on various cancers such as basal cell 13, hepatic 14, 15 and tongue squamous cell carcinoma 16, as well as cervical 17, breast 18, melanoma 19-21, gastric 22, 23, colon 24, lung 25 and ovarian 26 cancer, and that its short isoform (CASC15-S) acts as a tumor suppressor in neuroblastoma 44.

Nevertheless, the literature concerning the lncRNA CASC15 role in ovarian cancer has reported inconsistent results. The study conducted by Li et al 26 shows that CASC15 plays a tumor promotor role in ovarian cancers based on both clinical and basic data, which is consistent with our study. Meanwhile, another study conducted by Shi et al. 27 presents the opposite results, which reasoned low expression of CASC15 in tumor tissue compared to normal ovarian tissues, however, it seems to be controversial because recent studies strongly support that serous ovarian carcinoma develop from the fallopian tube epithelium 45-47.

Recently, molecular similarities have been revealed between gynecological and breast cancer in a comprehensive integrative molecular analysis of the complete 33 types of tumors in TCGA 48. Previous studies have suggested that lncRNA CASC15 exerts tumor-promoting function in cervical 17 and breast 18 cancer, which is prominently influenced by female hormones resembling ovarian cancer. Moreover, as shown in Figure 1B, forest plot indicated that high CASC15 expression correlated with worse OS (HR = 1.53, 95% CI: 1.09-2.15) for ovarian cancer patients in 8 completely independent TCGA and GEO cohorts. Combined with basic data, our research indicated that CASC15 plays a tumor promoter role in ovarian cancer.

Our *in vitro* and *in vivo* results indicated an association between CASC15 transcript level and ovarian cancer metastasis. In the present study, a series of experiments were performed to investigate the biological mechanism by which CASC15 exerted its function in ovarian cancer EMT and metastasis progression. Recently, CASC15 was identified as a new TGF-β-induced lncRNA in intrahepatic cholangiocarcinoma 49. Notably, our study found that CASC15 was a TGF-β downstream molecule and involved in the TGF-β/SMAD3 pathway in ovarian cancer: CASC15 was upregulated by TGF-β1, and then increased the expression of SMAD3 via serving as a ceRNA to facilitate cell migration and invasion in ovarian cancer. Our study discovered a novel interaction between lncRNA and miRNA cluster rather than an individual miRNA. MiR-23b-3p, miR-24-3p and miR-27b-3p compose the miR-23b cluster, but only miR-23b-3p and miR-24-3p, and not miR-27b-3p, targeted CASC15 mRNA directly. Previous studies revealed that miR-23b is an independent prognostic marker suppressing the progression of ovarian cancer 50, 51, while minimal work has been performed on the effect of miR-24-3p on ovarian cancer. We found that miR-23b-3p and miR-24-3p could additively suppress cancer metastasis through EMT in ovarian cancer cells independently of CASC15.

We cannot exclude the possibility that lncRNA CASC15 promoted ovarian cancer EMT progress and metastasis through means other than the miR-23b-3p/miR-24-3p/SMAD3 pathway. On one hand, apart from SMAD3 protein, CASC15 knockdown could decrease SMAD2 mRNA expression as well (Figure S4D). However, the miR-23b-3p/miR-24-3p-mediated SMAD2 expression decrease occurred only at the translational level, but not the transcriptional level (Figure S4E), suggesting that CASC15 utilized another approach to regulate SMAD2 mRNA expression, which also contributed to CASC15's tumor-promoting role. On the other hand, Jing et al. reported that CASC15 facilitates proliferation and metastasis of colon cancer through the Wnt/β-catenin signaling pathway activation 24; further research is needed to clarify the relationship between CASC15 and the Wnt/β-catenin pathway in ovarian cancer.

As a transcription factor, SMAD3 carries out its function via binding to the SBEs on the promoters of downstream effector genes and thus stimulating their transcriptional activities. We found, for the first time, that SMAD3 would permit the increased expression of CASC15. In this context, our results described a positive feedback loop of CASC15-SMAD3 in which CASC15 promoted the ovarian cancer EMT program and metastasis by increasing SMAD3 expression; in turn, SMAD3 stimulated the promoter activity of lncRNA CASC15.

## Supplementary Material

Supplementary methods, figures and tables.
