# Supplementary material for: LncRNA CASC15, MiR-23b Cluster and SMAD3 form a Novel Positive Feedback Loop to promote Epithelial-Mesenchymal Transition and Metastasis in Ovarian Cancer
Source: Int J Biol Sci. 2022 Feb 21;18(5):1989–2002. doi: 10.7150/ijbs.67486 (PMC8935218; doi:10.7150/ijbs.67486)
Supplement: Supplementary file 1 — Supplementary methods, figures and tables. [file ijbsv18p1989s1.pdf]

Supplementary Information

LncRNA CASC15, MiR-23b Cluster And SMAD3 Form A Novel Positive Feedback Loop To Promote Epithelial-Mesenchymal Transition And Metastasis In Ovarian Cancer

Hui Lin[1#], Xian Xu[1#], Kelie Chen[1#], Zhiqin Fu[2], Shengchao Wang[1], Yaqing Chen[2], Honghe Zhang[3], Yuequn Niu[1], Hanwen Chen[1,4], Hongfei Yu[3], Jian-zhong Shao[5], Weiguo Lu[1], Yihua Wu[1], Dajing Xia[1]

Corresponding: Dajing Xia (dxia@zju.edu.cn) and Yihua Wu (georgewu@zju.edu.cn).

Supplemental Text

Cell lines

SKOV3 were cultured in McCoy's 5A medium (Gibco, USA) and other cell lines were cultured in DMEM medium (Gibco, USA). Media were supplemented with 10% fetal bovine serum, 1% penicillin/streptomycin at 37 ℃ in a humidified atmosphere containing 5% CO_2. Cells in the logarithmic growth phase were used in subsequent experiments.

Cell migration

After cells reached 100% confluence, monolayer cells were wounded by scratching the surface straightly as uniformly as possible with a sterilized 200 μl pipette tip. Then the wells were rinsed three times with PBS and replaced with indicated serum-free media. Images were captured by a digital microscopy (Carl Zeiss Jena, Germany) at each indicated time. Experiments were performed in triplicate.

Cell invasion

The cell invasion assay was performed using a 24-well Transwell chamber (Corning, USA). Cells were harvested and suspended in 100 μl serum-free medium were seeded into the upper chamber with an 8-μm pore size insert pre-coated with Matrigel Matrix (BD Biosciences, USA). The lower chamber was filled with 600 μl medium containing 10% FBS. After incubation for 18 hours, cells were stained with a

0.4% crystal violet solution. The invading cells were imaged randomly using a digital microscopy (Carl Zeiss Jena, Germany). The number of cells were counted in 5 randomly selected fields. Experiments were performed in triplicate.

Western blots

The cells were collected and lysed with cell lysis buffer (Beyotime, China). Samples of the lysates were separated on 7%–13.5% SDS-PAGE gels and transferred to nitrocellulose filter membranes. Membranes were incubated overnight with primary antibodies, including ZEB1 (1:1000, Cell signaling Technology, CST), E-Cadherin (1:1000, CST), N-Cadherin (1:1000, CST), Slug (1:1000, CST), Snail (1:1000, CST), Claudin-1 (1:1000, CST), SMAD3 (1:1000, CST), HNF1B (1:1000, Abcam), PARP (1:1000, CST), pro-caspase-3 (1:1000, CST), cleaved caspase-3 (1:1000, CST), GAPDH (1:5000, CST) and β-actin (1:5000, CST). The next day, membranes were incubated with a secondary horseradish peroxidase-conjugated anti-rabbit or anti-mouse IgG antibody, and then specific bands were visualized by ChemiScope 3300 Mini (Clinx, China) using the ECL substrate (Cyanagen, Italy). GAPDH or β-actin was used as loading control. Experiments were performed in triplicate.

Quantitative real-time PCR

Total RNA was extracted from cells using Trizol reagent (Sangon Biotech, China) according to the manufacturer's instructions. For mRNA quantification, RNA was

reverse transcribed to cDNA using PrimeScript™ RT reagent Kit with gDNA Eraser (Takara, Japan). Quantitative real-time PCR was performed using cDNA primers specific for mRNA. The gene GAPDH was used as internal control. For miRNA quantification, reverse transcription was performed using Mir-X™ miRNA First Strand Synthesis Kit (Takara, Japan). MiRNA specific 5’ primers and mRQ 3’ primer was used during quantitative real-time PCR. The gene U6 was used as an internal control. The mRNA and miRNA primer sequences were provided in Table S3, S4, respectively. All the real-time PCR reactions were performed using Takara’s SYBR Premix Ex Taq™ II (Tli RNaseH Plus) in the Applied Biosystems 7500 Fast Real-Time PCR System (Applied Biosystems, USA). The $2^{-\Delta\Delta Ct}$ method was used for quantification and fold change for target genes was normalized by internal control. Experiments were performed in triplicate.

Luciferase reporter assay

For 3' UTR luciferase reporter assays, HEK-293T and SKOV3 cells were co-transfected with miR-23b-3p, miR-24-3p or miR-27b-3p mimics or negative control and indicated pmirGLO-3' UTR wide-type or mutant plasmids using Lipofectamine 3000 reagent (Thermo Fisher Scientific, USA).

For luciferase reporter assay to measure promoter activities, indicated cells were cotransfected with pGL4.20-CASC15/SMAD3 promoter fragment, pRL-TK Renilla

luciferase reporter, and pcDNA3.1-SMAD3 or siRNAs specific for SMAD3/HNF1B using Lipofectamine 3000 reagent (Thermo Fisher Scientific, USA).

48 h post transfection, Firefly luciferase activities and *Renilla* luciferase activities were measured using the dual-luciferase reporter assay system (Promega, USA). Firefly luciferase activity was normalized to *Renilla* activity and presented as relative luciferase activity. Experiments were performed in triplicate.

In vivo tumor growth in xenograft model

SKOV3 cells stably transfected with sh-CASC15 or sh-NC were constructed in our laboratory. All the transfected SKOV3 cells were then labeled with pHIV-Luciferase. The *in vivo* ovarian xenograft model was performed using 5-week-old female NOD/SCID mice. The NOD/SCID mice were divided into 2 groups randomly and were administered 100 μl 1×10^7/1 ml indicated cells by intraperitoneal injection (i.p.). Bioluminescent flux (photons/s/sr/cm^2) was determined on week 1, 3, and 5 to assess tumor foci in the abdomen using the IVIS spectrum imaging system (Caliper, Newton, USA) and Living Image software (Caliper, Newton, USA). The mice were sacrificed 6 weeks after injection. The study was approved by the Ethics Committee of Zhejiang University.

Computational analyses and bioinformatics

The CASC15 expression data of ovarian cancer patients of GEO (Gene Expression Omnibus) dataset, were downloaded from an international public repository Gene Expression Omnibus [1-8] (GEO, https://www.ncbi.nlm.nih.gov/geo/, last accessed November 15, 2020). And the CASC15, SMAD3 expression data of ovarian cancer specimens of The Cancer Genome Atlas (TCGA) were extracted from exon expression dataset download from UCSC Cancer Browser [9] (http://genome.ucsc.edu/, last accessed November 15, 2020), which was a suite of web-based tools to visualize, integrate and analyze cancer genomics and associated clinical data. The expression quantification was done by averaging the expression of its exons.

Potential miRNA targets on CASC15, SMAD3 and HNF1B mRNAs were predicted by the computer algorithm TargetScanHuman 7.2 [10] (http://www.targetscan.org/vert_72/, last accessed November 15, 2020), DIANA tools [11, 12] (http://carolina.imis.athena-innovation.gr/diana_tools/web/index.php, last accessed November 15, 2020) and starBase v3.0 [13] (http://starbase.sysu.edu.cn/, last accessed November 15, 2020). The mature miRNA sequences used in this study were download from miRBase [14] (http://www.mirbase.org/, last accessed November 15, 2020).

To identify putative transcription factor binding sites in DNA sequences, we analyzed the SMAD3 and CASC15 promoter in PROMO (TRANSFAC v8.3) [15] (http://alggen.lsi.upc.es/cgi-bin/promo_v3/promo/promoinit.cgi?dirDB=TF_8.3, last

accessed November 15, 2020) and JASPAR database [16] (http://jaspar.genereg.net, last accessed November 15, 2020).

Statistical analyses

Statistical analyses were performed using GraphPad Prism 8 software (GraphPad Software Inc.). All data were expressed as the mean ± standard error of mean (SEM) of at least three independent experiments. Differences between the two groups were assessed using Student's t-test. We adopted one-way analysis of variance for multiple comparisons. A value of $p < 0.05$ was considered statistically significant unless otherwise specified.

The Kaplan–Meier method with log-rank testing was used in survival analysis. Scatter plots were created by P package ggpubr. Pearson correlation coefficients with *p* values were calculated. Heterogeneity between individual studies was assessed by χ^2 test and I^2 test; $p \leqslant 0.05$ and/or $I^2 > 50\%$ indicates significant heterogeneity. Summary HRs (RRs) and 95% CI were calculated using a random-effects model when the heterogeneity was significant, and a fixed-effects model was applied otherwise. Two-sided *p* values were calculated, with a *p* value <0.05 considered significant for all tests.

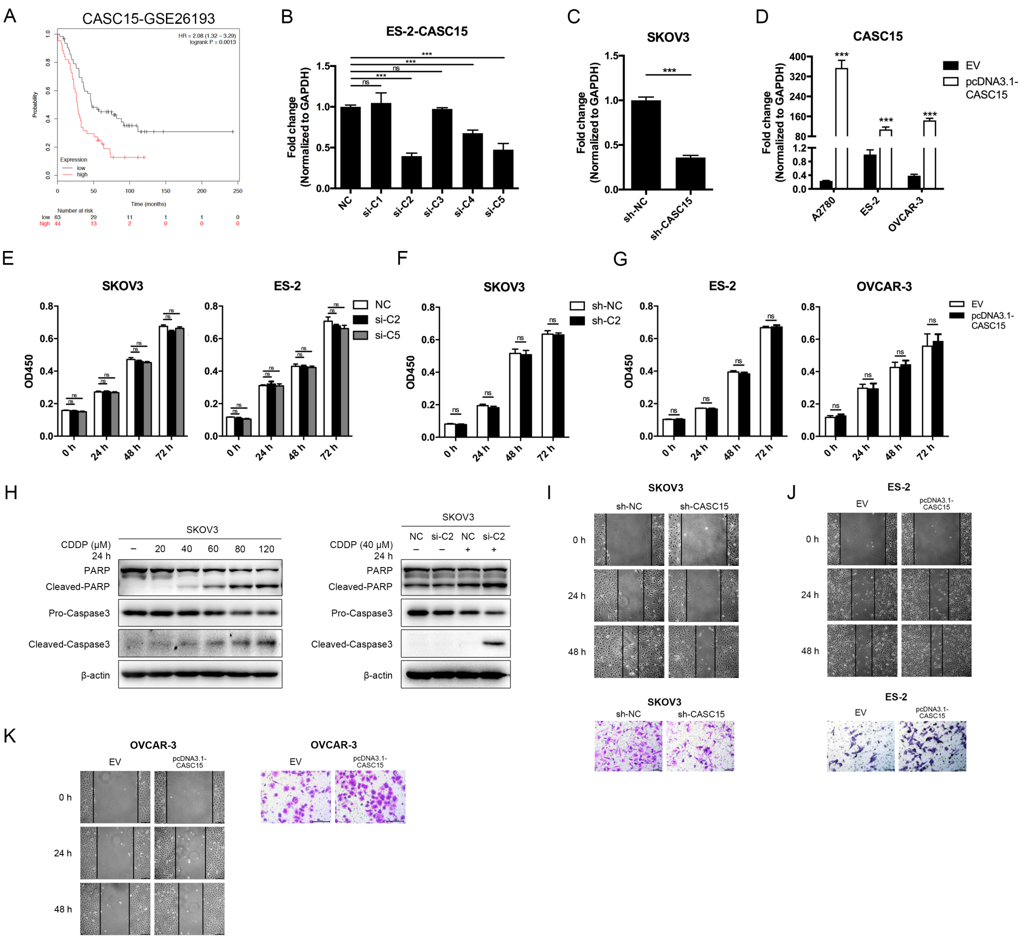

Figure S1. CASC15 promoted ovarian cancer cell migration and invasion. (A) Kaplan-Meier overall survival curves for ovarian cancer patients with high and low CASC15 expression from GEO database GSE26193. (B) Relative expression of CASC15 in ES-2 cells 48 h post CASC15 siRNA transfection by RT-qPCR. (C) Relative expression of CASC15 in pLKO.1-sh-CASC15 and pLKO.1-sh-NC transfected SKOV3 cells by RT-qPCR. (D) Relative expression of CASC15 in pcDNA3.1-CASC15 transfected A2780, ES-2 and OVCAR-3 cells by RT-qPCR. (E) CCK-8 proliferation assay in si-NC or si-C2/si-C5 transfected SKOV3 and ES-2 cells. (F) CCK-8 proliferation assay in pLKO.1-sh-CASC15 and pLKO.1-sh-NC transfected SKOV3 cells. (G) CCK-8 proliferation assay in pcDNA3.1-CASC15 transfected ES-2/OVCAR-3 cells. (H) Expression of PARP, Pro-Caspase-3 and Cleaved-Caspase-3 in si-NC or si-C2 transfected, cisplatin treated SKOV3 cells. (I) Wound scratch assay (upper) and transwell invasion assay (lower) in pLKO.1-sh-CASC15 and pLKO.1-sh-NC transfected SKOV3 cells. (J)

Wound scratch assay (upper) and transwell invasion assay (lower) in pcDNA3.1-CASC15 transfected ES-2 cells. (K) Wound scratch assay (left) and transwell invasion assay (right) in pcDNA3.1-CASC15 transfected OVCAR-3 cells. GAPDH serves as the internal control in RT-qPCR, and β-actin serves as the internal control in western blots. Scale bar, 50 μM. ns, no statistical significance, * p <0.05, ** p <0.01, *** p <0.001. Values are mean ± SEM. Data are representative of three independent experiments.

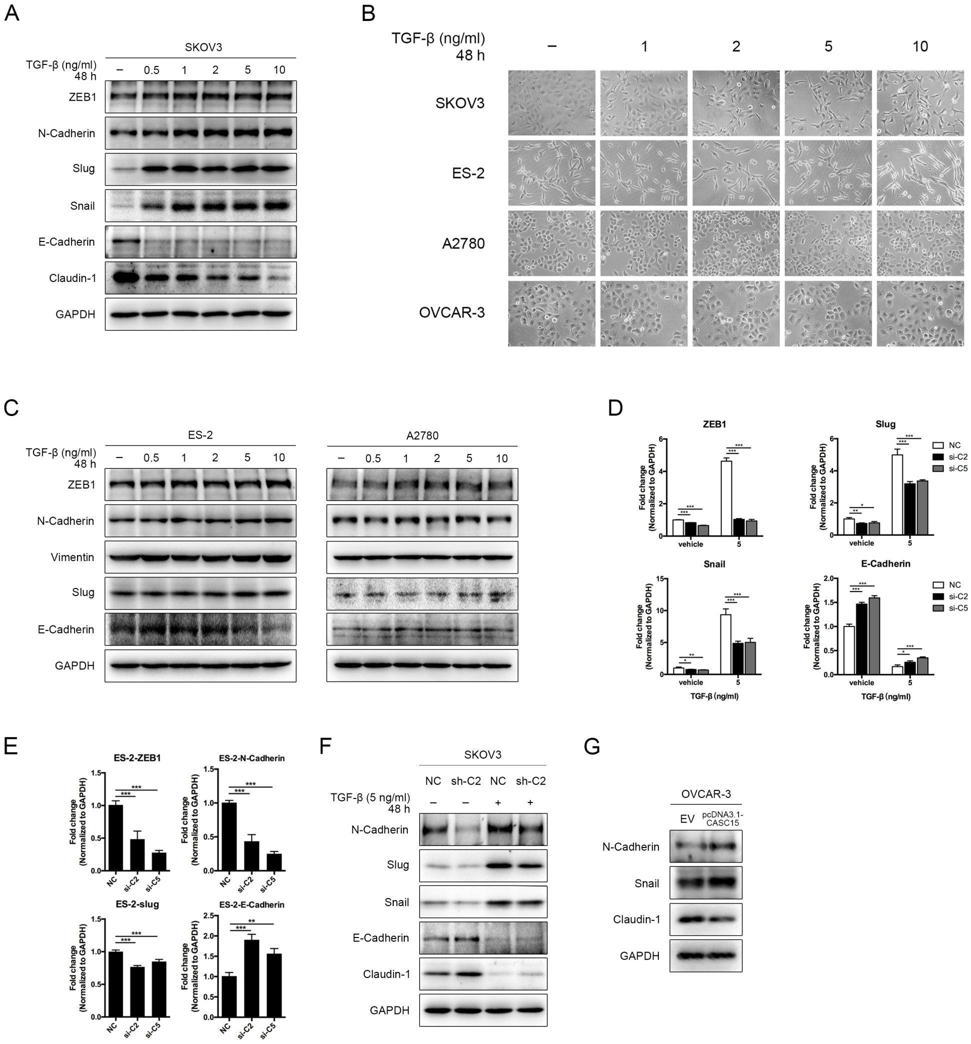

Figure S2. CASC15 was upregulated by TGF-β and triggered EMT in ovarian cancer cells. (A) Expression of ZEB1, N-Cadherin, Slug, Snail, E-Cadherin and Claudin-1 in TGF-β treated SKOV3 cells as determined by western blots. (B) Morphology of TGF-β treated SKOV3, ES-2, A2780 and OVCAR-3 cells. (C) Expression of EMT markers in TGF-β treated ES-2 and A2780 cells as determined by western blots. (D) mRNA levels of ZEB1, Slug, Snail and E-Cadherin in si-NC or si-C2/si-C5 transfected, TGF-β treated SKOV3 cells by RT-qPCR. (E) mRNA levels of ZEB1, N-Cadherin, Slug, and E-Cadherin in si-NC or si-C2/si-C5 transfected ES-2 cells by RT-qPCR. (F) Western blots of N-Cadherin, Slug, Snail, E-Cadherin, and Claudin-1 in TGF-β or vehicle treated pLKO.1-sh-CASC15 and pLKO.1-sh-NC transfected SKOV3 cells. (G) Western blots of N-Cadherin, Snail and Claudin-1 in pcDNA3.1-CASC15 transfected

OVCAR-3 cells. GAPDH serves as the internal control in RT-qPCR and western blots. ns, no statistical significance, * p <0.05, ** p <0.01, *** p <0.001. Values are mean ± SEM. Data are representative of three independent experiments.

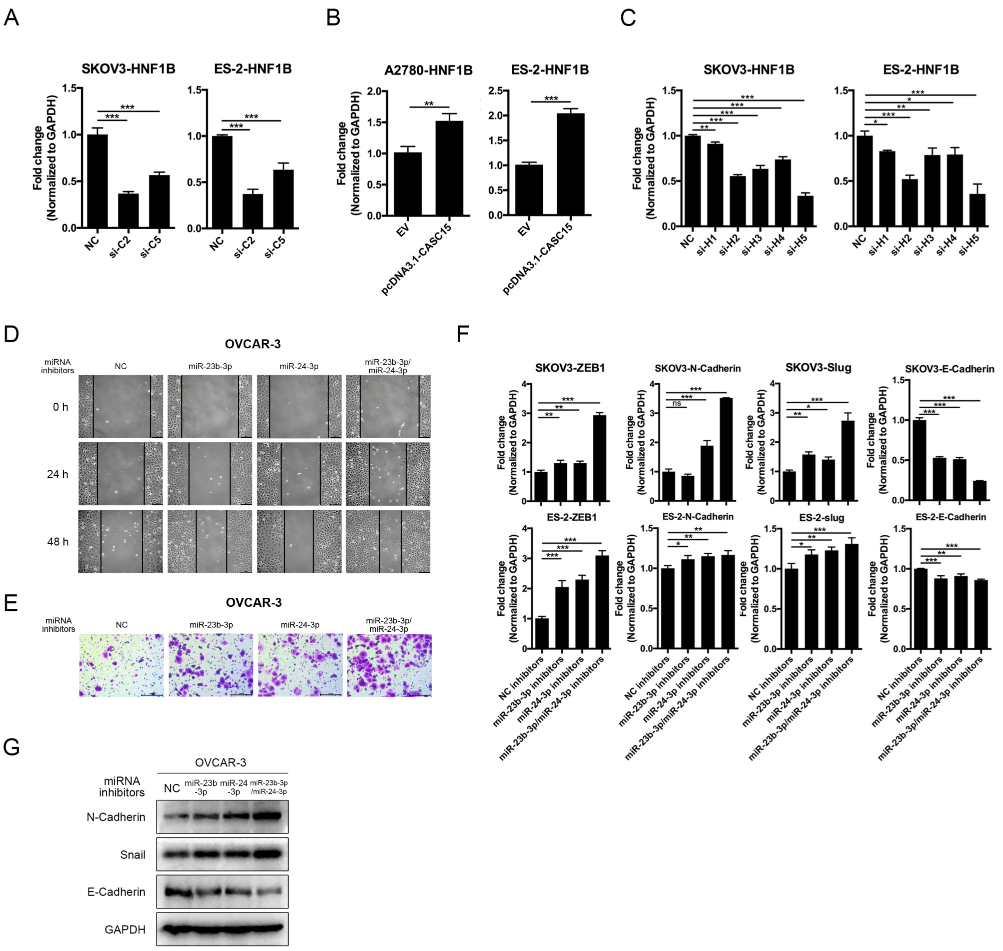

Figure S3. MiR-23b-3p and miR-24-3p additively suppressed EMT and metastasis, and rescued CASC15's promoting effect on ovarian cancer cells. (A) RT-qPCR analysis of HNF1B expression in si-NC or si-C2/si-C5 transfected SKOV3 and ES-2 cells. (B) RT-qPCR analysis of HNF1B expression in pcDNA3.1-CASC15 transfected A2780 and ES-2 cells. (C) Relative expression of HNF1B in SKOV3 and ES-2 cells 48 h post HNF1B siRNA transfection by RT-qPCR. (D) Representative images of the wound scratch assay in a 100× light microscope utilizing the OVCAR-3 cell lines 24 and 48 h after scratching. Cells were transfected with miR-23b-3p or miR-24-3p inhibitors alone, or miR-23b-3p and miR-24-3p inhibitors together. (E) Representative digital images of transwell invasion assay in OVCAR-3 cells after transfection with miR-23b-3p or miR-24-3p inhibitors alone, or miR-23b-3p and miR-24-3p inhibitors together. (F) mRNA levels of ZEB1, N-Cadherin, Slug, and E-Cadherin in miR-23b-3p or miR-24-3p

inhibitor transfected SKOV3 and ES-2 cells by RT-qPCR. (G) Western blots of EMT markers in OVCAR-3 cells after transfection with miR-23b-3p or miR-24-3p inhibitors alone, or miR-23b-3p and miR-24-3p inhibitors together. GAPDH serves as the internal control in RT-qPCR and western blots. Scale bar, 50 μM. ns, no statistical significance, * p <0.05, ** p <0.01, *** p <0.001. Values are mean ± SEM. Data are representative of three independent experiments.

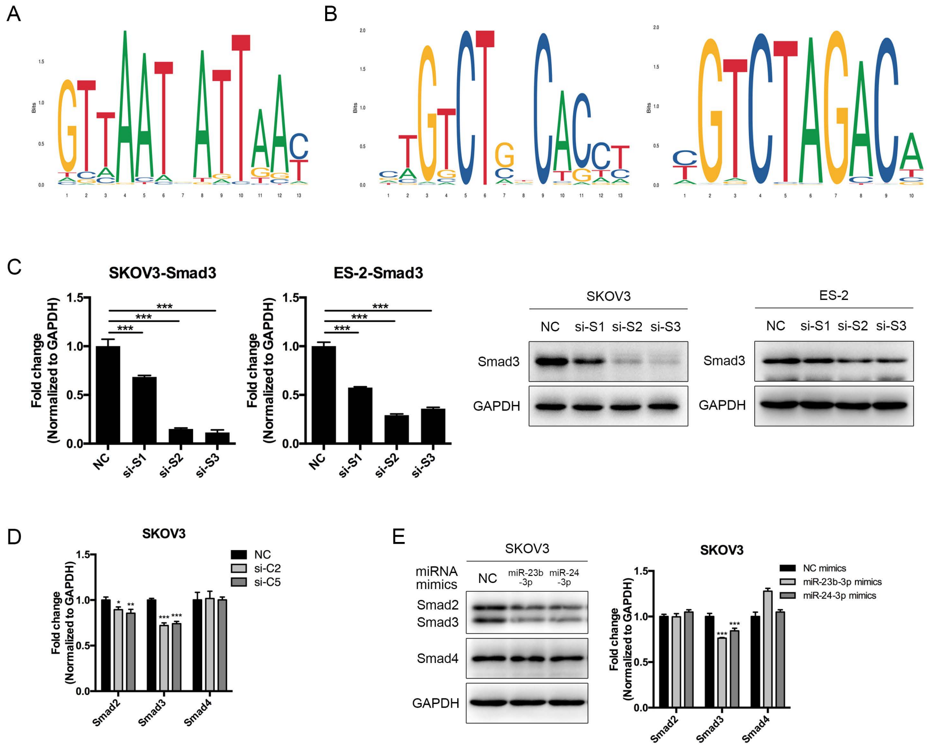

Figure S4. CASC15 didn't upregulate SMAD2 expression through miR-23b-3p/miR-24-3p sequestration. (A) The sequence logo of the HNF1B position frequency matrix in the JASPAR database. (B) The sequence logos of the SMAD3 position frequency matrix SMAD2::SMAD3::SMAD4 (left) and SMAD3 (right) in the JASPAR database. (C) Expression of SMAD3 in SKOV3 and ES-2 cells 48 h post SMAD3 siRNA transfection as determined by RT-qPCR and western blots. (D) Expression of SMAD2, SMAD3, and SMAD4 in si-NC or si-C2/si-C5 transfected SKOV3 cells as determined by RT-qPCR. (E) Expression of SMAD2, SMAD3, and SMAD4 in miR-23b-3p or miR-24-3p mimics transfected SKOV3 cells as determined by RT-qPCR and western blots. GAPDH serves as the internal control in RT-qPCR and western blots. ns, no statistical significance, * p <0.05, ** p <0.01, *** p <0.001. Values are mean ± SEM. Data are representative of three independent experiments.

Table S1. List of siRNA sequences.

siRNA	sense (5'→3')	antisense (5'→3')
Negative control	UUCUCCGAACGUGUCACGUTT	ACGUGACACGUUCGGAGAATT
Si-CASC15-1	GACAGUCAGAAUGACUGAUTT	AUCAGUCAUUCUGACUGUCTT
Si-CASC15-2	GAUUUGUCCAGGAGCAGAUTT	AUCUGCUCCUGGACAAAUCTT
Si-CASC15-3	GAAGUACCCUCAGGUGACUTT	AGUCACCUGAGGGUACUUCTT
Si-CASC15-4	GGGAAUUCUCCACCUUAAATT	UUUAAGGUGGAGAAUUCCCTT
Si-CASC15-5	GUGACUACAGAUGUGUUAATT	UUAACACAUCUGUAGUCACTT
Si-HNF1B-1	AUCACUUCCUCCUCAACAAUC	GAUUGUUGAGGAGGAAGUGAU
Si-HNF1B-2	AGUCAGCACCUUGACGAAUAU	AUAUUCGUCAAGGUGCUGACU
Si-HNF1B-3	ACAGCCUCUCCCACCAUAAUC	GAUUAUGGUGGGAGAGGCUGU
Si-HNF1B-4	CCGUACUGUCUAUGUUGUGAU	AUCACAACAUAGACAGUACGG
Si-HNF1B-5	CAGUCCAGAGUUCUGGAAAUA	UAUUUCCAGAACUCUGGACUG
Si-SMAD3-1	CCCAGCACAUAAUAACUUGGA	UCCAAGUUAUUAUGUGCUGGG
Si-SMAD3-2	UCCGCAUGAGCUUCGUCAAAG	CUUUGACGAAGCUCAUGCGGA
Si-SMAD3-3	GAGCCUGGUCAAGAAACUCAA	UUGAGUUUCUUGACCAGGCUC

Table S2. List of shRNA sequences.

shRNA	sense (5'→3')
sh-NC	CCTAAGGTTAAGTCGCCCTCGCTCGAGCGAGGGCGACTTAACCTTAGG
sh-CASC15	AAGATTTGTCCAGGAGCAGATCTCGAGATCTGCTCCTGGACAAATCTT

Table S3. List of mRNA primer sequences.

Target gene	Forward primer (5'→3')	Reverse primer (5'→3')
CASC15	TCCAAGTGTGACTGCCAAGA	CATTTCCTCTGGGTTTTCCA
ZEB1	AAGTGGCGGTAGATGGTAATGT	AAGGAAGACTGATGGCTGAAAT
N-Cadherin	ATGGGAAATGGAAACTTGATGGC	TGGAAAGCTTCTCACGGCAT
Slug	AACAGTATGTGCCTTGGGGG	AAAAGGCACTTGGAAGGGGT
Snail	CTCGGACCTTCTCCCGAATG	AAAGTCCTGTGGGGCTGATG
E-Cadherin	ACAGCACGTACACAGCCCTA	GCAGAAGTGTCCCTGTTCCAG
Claudin-1	TTTACTCCTATGCCGGCGAC	GAGGATGCCAACCACCATCA
SMAD3	AGGGCTTTGAGGCTGTCTACC	GTGCTGGTCACTGTCTGTCTCCT
HNF1B	CCTCCGACAGTTCAGTCAACA	CTTGCTGGGGTTCTTTTGCC
SMAD2	GCCTTTACAGCTTCTCTGAACAA	ATGTGGCAATCCTTTTCGAT
SMAD4	GCTGCAGAGCCCAGTTTAGA	CCCCAAAGCAGAAGCTACGA
GAPDH	CACCCACTCCTCCACCTTTG	CCACCACCCTGTTGCTGTAG

Table S4. List of microRNA primer sequences.

microRNA	3' specific primer (5'→3')
hsa-miR-23b-3p	ATCACATTGCCAGGGATTACCAC
hsa-miR-24-3p	TGGCTCAGTTCAGCAGGAACAG
hsa-miR-27b-3p	TTCACAGTGGCTAAGTTCTGC

MicroRNA specific 5' primer, U6-Forward and U6-Reverse were provided by the Mir-X™ miRNA First Strand Synthesis Kit (Takara, Japan).

Table S5. Schema of miR-23b-3p, miR-24-3p and miR-27b-3p binding sites in predicted target 3' UTR sequences of human genes.

Gene	microRNA	Target Site	3' UTR Position
CASC15	miR-23b-3p	3' CCAU-- UA--GGGACCGUUACACUA 5' (miR-23b-3p)<br>5' ···GGUACAGGUGUAAAUGCUGUUCAUGUGAU··· 3' (3' UTR-WT)<br>5' ···GGTACACCATTAAAGGGACTTCTACACTA··· 3' (3' UTR-MUT)	chr6:22147894-22147916
CASC15	miR-24-3p	3' GACAA--GGAC----GACU-----U-----GACUCGGU 5' (miR-24-3p)<br>5' ···GGUUGCCAAAACUGACAUUGUCUGAGCCC··· 3' (3' UTR-WT)<br>5' ···GCAAGGGAAAAGACT CATTGT GACTCGGC··· 3' (3' UTR-MUT)	chr6:22149601-22149627
CASC15	miR-27b-3p	3' CGUCUUGAA---UC---GGUCACACUU 5' (miR-27b-3p)<br>5' ···UGCAUGCAGAAUUUUCUUUUUACUGUGAA··· 3' (3' UTR-WT)<br>5' ···TGCATCGTCTTGAATCTTTGGTCACACTT··· 3' (3' UTR-MUT)	chr6:22147507-22147530
SMAD3	miR-23b-3p	3' CCA---UUA---GGGACCGUUACACUA 5' (miR-23b-3p)<br>5' ···UGGCAGGUUAUGAACUUUG----AAUGUGAU··· 3' (3' UTR-WT)<br>5' ···TGGCACCATATGAAGGGAC----TTACACTA··· 3' (3' UTR-MUT)	chr15:67485127-67485148
HNF1B	miR-23b-3p	3' CCAU----UAGGGACCGUUACACUA 5' (miR-23b-3p)<br>5' ···GAAAGCCGUACUGUCUAUG---U---UGUGAU··· 3' (3' UTR-WT)<br>5' ···GAAAGCCCATCTTAGTAAC---T---ACACTA··· 3' (3' UTR-MUT)	chr17:36047060-36047078
HNF1B	miR-24-3p	3' GACAAGGACGACUUGACUCGGU 5' (miR-24-3p)<br>5' ···GAAGGACAUGU-G-CUAUUGAACUGAGCCA··· 3' (3' UTR-WT)<br>5' ···GAAGGACAACA-G-GAAGACTTGACTCGGT··· 3' (3' UTR-MUT)	chr17:36046978-36046997

Putative binding sites were mutated and highlighted in red.

Table S6. The predicted HNF1B-binding sites on the SMAD3 promoter.

	Matrix Name	Predicted sequence	Start	End
HNF1B-binding site 1	HNF1B	TTTTTTTTTAAC	-1430	-1419
HNF1B-binding site 2	HNF1B	GTTAAAAACAAAC	-1387	-1375
HNF1B-binding site 3	HNF1B	TTCAAAGATAAAT	-1033	-1021

Table S7. The predicted SMAD3-binding sites on the CASC15 promoter.

	Matrix Name	Predicted sequence	Start	End
SMAD3-binding site 1	SMAD2::SMAD3::SMAD4	GCGGCTCACACCC	-1985	-1973
SMAD3-binding site 2	SMAD3	CGTCCAGACT	-1867	-1858
SMAD3-binding site 3	SMAD2::SMAD3::SMAD4	CCGTCTACCTCAT	-1397	-1385

References

1. Barrett T, Wilhite SE, Ledoux P, Evangelista C, Kim IF, Tomashevsky M, et al. NCBI GEO: archive for functional genomics data sets-update. Nucleic Acids Research. 2013; 41: D991-D5.
2. Tothill RW, Tinker AV, George J, Brown R, Fox SB, Lade S, et al. Novel molecular subtypes of serous and endometrioid ovarian cancer linked to clinical outcome. Clin Cancer Res. 2008; 14: 5198-208.
3. Mateescu B, Batista L, Cardon M, Gruosso T, de Feraudy Y, Mariani O, et al. miR-141 and miR-200a act on ovarian tumorigenesis by controlling oxidative stress response. Nat Med. 2011; 17: 1627-35.
4. King ER, Tung CS, Tsang YT, Zu Z, Lok GT, Deavers MT, et al. The anterior gradient homolog 3 (AGR3) gene is associated with differentiation and survival in ovarian cancer. Am J Surg Pathol. 2011; 35: 904-12.
5. Ferriss JS, Kim Y, Duska L, Birrer M, Levine DA, Moskaluk C, et al. Multi-gene expression predictors of single drug responses to adjuvant chemotherapy in ovarian carcinoma: predicting platinum resistance. PLoS One. 2012; 7: e30550.
6. Konstantinopoulos PA, Spentzos D, Karlan BY, Taniguchi T, Fountzilas E, Francoeur N, et al. Gene expression profile of BRCAness that correlates with responsiveness to chemotherapy and with outcome in patients with epithelial ovarian cancer. J Clin Oncol. 2010; 28: 3555-61.
7. Lisowska KM, Olbryt M, Dudaladava V, Pamula-Pilat J, Kujawa K, Grzybowska E, et al. Gene expression analysis in ovarian cancer - faults and hints from DNA microarray study. Front Oncol. 2014; 4: 6.
8. Mok SC, Bonome T, Vathipadiekal V, Bell A, Johnson ME, Wong KK, et al. A gene signature predictive for outcome in advanced ovarian cancer identifies a survival factor: microfibril-associated glycoprotein 2. Cancer Cell. 2009; 16: 521-32.
9. Kent WJ, Sugnet CW, Furey TS, Roskin KM, Pringle TH, Zahler AM, et al. The human genome browser at UCSC. Genome Research. 2002; 12: 996-1006.
10. Agarwal V, Bell GW, Nam JW, Bartel DP. Predicting effective microRNA target sites in mammalian mRNAs. Elife. 2015; 4.
11. Paraskevopoulou MD, Georgakilas G, Kostoulas N, Vlachos IS, Vergoulis T, Reczko M, et al. DIANA-microT web server v5.0: service integration into miRNA functional analysis workflows. Nucleic Acids Research. 2013; 41: W169-W73.
12. Paraskevopoulou MD, Georgakilas G, Kostoulas N, Reczko M, Maragkakis M, Dalamagas TM, et al. DIANA-LncBase: experimentally verified and computationally predicted microRNA targets on long non-coding RNAs. Nucleic Acids Research. 2013; 41: D239-D45.
13. Li JH, Liu S, Zhou H, Qu LH, Yang JH. starBase v2.0: decoding miRNA-ceRNA, miRNA-ncRNA and protein-RNA interaction networks from large-scale CLIP-Seq data. Nucleic Acids Research. 2014; 42: D92-D7.
14. Kozomara A, Griffiths-Jones S. miRBase: annotating high confidence microRNAs using deep sequencing data. Nucleic Acids Research. 2014; 42: D68-D73.
15. Messeguer X, Escudero R, Farre D, Nunez O, Martinez J, Alba M. PROMO: detection of known transcription regulatory elements using species-tailored searches. Bioinformatics. 2002; 18: 333-4.
16. Khan A, Fornes O, Stigliani A, Gheorghe M, Castro-Mondragon JA, van der Lee R, et al. JASPAR 2018: update of the open-access database of transcription factor binding profiles and its web framework. Nucleic Acids Research. 2018; 46: D260-D6.
